# On Nomological Validity and Auxiliary Assumptions: The Importance of Simultaneously Testing Effects in Social Cognitive Theories Applied to Health Behavior and Some Guidelines

**DOI:** 10.3389/fpsyg.2017.01933

**Published:** 2017-11-03

**Authors:** Martin S. Hagger, Daniel F. Gucciardi, Nikos L. D. Chatzisarantis

**Affiliations:** ^1^Health Psychology and Behavioural Medicine Research Group, School of Psychology, Faculty of Health Sciences, Curtin University, Perth, WA, Australia; ^2^School of Physiotherapy and Exercise Science, Faculty of Health Sciences, Curtin University, Perth, WA, Australia

**Keywords:** nomological validity, predictive validity, falsifiability, path analysis, meta-analysis, replication, auxiliary assumptions

## Abstract

Tests of social cognitive theories provide informative data on the factors that relate to health behavior, and the processes and mechanisms involved. In the present article, we contend that tests of social cognitive theories should adhere to the principles of nomological validity, defined as the degree to which predictions in a formal theoretical network are confirmed. We highlight the importance of nomological validity tests to ensure theory predictions can be disconfirmed through observation. We argue that researchers should be explicit on the conditions that lead to theory disconfirmation, and identify any auxiliary assumptions on which theory effects may be conditional. We contend that few researchers formally test the nomological validity of theories, or outline conditions that lead to model rejection and the auxiliary assumptions that may explain findings that run counter to hypotheses, raising potential for ‘falsification evasion.’ We present a brief analysis of studies (*k* = 122) testing four key social cognitive theories in health behavior to illustrate deficiencies in reporting theory tests and evaluations of nomological validity. Our analysis revealed that few articles report explicit statements suggesting that their findings support or reject the hypotheses of the theories tested, even when findings point to rejection. We illustrate the importance of explicit *a priori* specification of fundamental theory hypotheses and associated auxiliary assumptions, and identification of the conditions which would lead to rejection of theory predictions. We also demonstrate the value of confirmatory analytic techniques, meta-analytic structural equation modeling, and Bayesian analyses in providing robust converging evidence for nomological validity. We provide a set of guidelines for researchers on how to adopt and apply the nomological validity approach to testing health behavior models.

## Introduction

Testing the validity of social cognitive models applied to the prediction of health behavior provides an important evidence base to inform the development of behavioral interventions aimed at promoting health behavior (Biddle et al., 2007; Weinstein, 2007; Leventhal et al., 2008; Schwarzer, 2008; Conner and Norman, 2015; Sniehotta et al., 2015). Model tests assist in identifying the manipulable factors that can then be targeted by behavioral strategies and techniques that form the content of interventions. Testing the adequacy of models in explaining health-related outcomes, therefore, has high *translational* value (Moss-Morris and Yardley, 2008; Wallace et al., 2014).

Theoretical models in psychology provide representations of the factors and processes that relate to outcomes of interest. The models are often complex involving multiple antecedent, mediator, and outcome variables and proposals for the pattern of relations among them in a *nomological network*. The specification of models in this way is consistent with the Popper’s (1959) position that social scientists should seek to provide a comprehensive description of the situational and interpersonal factors affecting human action. Central to this position is the requirement that such descriptions are specified in advance of observation and should be verified or disconfirmed through rigorous empirical tests. Such an approach requires clear *a priori* specification of sets of relations among social cognitive variables as antecedents, mediators and consequents in the nomological network followed by subsequent simultaneous tests of the network. This position was advocated by Cronbach and Meehl (1955), among others, when proposing the importance of subjecting theories to strict tests of their nomological validity, and abandoning the theory or proposing modifications and subsequent tests in an iterative approach.

Of course, a strict approach to theory falsification and nomological validity, in the Popperian sense, has been noted as problematic. Following Popper’s original assertion, Lakatos (1978) noted that failures to support or confirm a theoretical prediction, or simultaneous set of predictions in a nomological network, could be proposed as evidence for the falsification of the theory. But it could also be attributed to a number of conditions or assumptions that lie outside the theory which may explain the failure to support the prediction. These *auxiliary assumptions* represent other external conditions that may affect conclusions about the hypothesized relations among theory variables, if these conditions are not measured and confirmed directly during the analysis (Trafimow, 2012). The potential to explain away failures to falsify theory predictions by auxiliary assumptions renders absolute deductive falsification impossible: the failure could be attributed to the theory or auxiliary assumptions. An approach to deal with this problem is to propose theories that make ‘risky’ predictions, that is, predictions that would be false if the theory was untrue, and to explicitly state how theoretical predictions are expected to be affected by auxiliary assumptions (Trafimow, 2012, 2017).

Theory predictions, therefore, should be accompanied by additional hypotheses or statements representing the auxiliary assumptions and how they affect predictions. In this way, falsification is not abandoned, rather strict falsification is replaced with a more nuanced version: ‘reasonable falsification’ (Trafimow, 2009). On this basis, if risky predictions can be shown to be incorrect under conditions of proposed auxiliary assumptions, then the researcher can claim a level of falsifiability of the theory, bearing in mind the caveat that other auxiliary assumptions can be proposed. Taken together, conducting multiple rigorous tests of risky predictions under multiple auxiliary assumptions will provide converging evidence for the theory. Accordingly, Trafimow (2009) notes: “Ultimately, when one decides how much to believe or disbelieve a theory, the issue is the weight of the evidence, the plausibility of alternative explanations, presumptions about the validity of auxiliary assumptions, and so on, rather than conclusive proof or disproof” (p. 505).

An illustrative example comes from the recent debate on the ego-depletion effect and the ‘strength’ model of self-control (Carter and McCullough, 2014; Baumeister and Vohs, 2016; Hagger and Chatzisarantis, 2016a; Lurquin and Miyake, 2017). In the strength model, the proposition that: “if people exert effort on an initial self-control task (antecedent) then performance on a subsequent self-control task will decline (consequent)” is predicted to be true. According to Popper (1959), to disconfirm this proposition the experimenter will need to (i) confirm the antecedent (the ‘if’) and so show experimentally that people exert effort on an initial self-control task and (ii) show that performance on the second task does not decline (the ‘then’). But many other factors are known to affect individuals’ willingness to exert effort in the initial task and these represent auxiliary assumptions regarding the strength model of self-control (e.g., Allom et al., 2016; Dang, 2016; Lee et al., 2016). Consistent with Lakatos’ (1978) propositions, experiments should be devised to show (i) disconfirmation when the factors are present and (ii) confirmation if factors are present. The theory is disconfirmed if findings of the tests are always inconsistent with theory until also additional factors or auxiliary assumptions have been accounted for. Similarly, Trafimow (2009) proposes that “one must try out all possible combinations of auxiliary assumptions in conjunction with the theory and show that, in every case, the resulting prediction cannot be tested, even in principle” (p. 504). He illustrates his point in tests of the distinction between attitudinal and normative beliefs in the theory of planned behavior under different assumptions (Trafimow, 2009). Taken together, these examples illustrate the importance of accounting for auxiliary assumptions when testing risky predictions in theories in psychology.

Extending these arguments, we contend in this article that tests of social cognitive theories applied in health behavior contexts have tended to focus on testing predictions of individual relations within theories, and fail to formally test the validity of the predictions of theory as a whole. This practice has rendered many of the tests problematic as means to provide evidence in support of the predictions of the theory. Such practice has led researchers to claim support for theoretical predictions, when they have, in fact, failed to test the theory. As a consequence, such tests do not contribute to the converging evidence for the validity of the theory. If one assumes that evidence supporting a theory is built on multiple supportive tests over time of its hypothesized predictions specified *a priori*, that is, its nomological validity, and accompanying assumptions, then revealing that many tests fail to support the theory, under the principle of reasonable falsification, undermines the strength of the evidence. A further problem is that researchers have tended to attribute failed tests of individual predictions within a nomological network to conditions or variables that lie outside the theory, that is, auxiliary assumptions, and tended to claim support for the prediction of the theory as a whole. Such dismissal is problematic from the standpoint of reasonable falsifiability given that the auxiliary assumptions are often specified *post hoc*, nor do they feature in the tests. This renders the evidence unhelpful in terms of contributing evidence in support for the predictions of the theory.

In this article we outline how rigorous nomological validity tests are essential for adequate evaluation of social cognitive theories applied in the health behavior domain, that researchers often inadequately specify their predictions and associated assumptions prior to testing the theories, and, as a result, the body of evidence supporting social cognitive theories may be weaker than previously thought. Failure to adequately specify the sets of hypotheses that comprise a theory, that is, the nomological network, and the associated conditions that may affect the predictions, that is, the auxiliary assumptions, potentially opens the door to *ad hoc* explanations for findings that run counter to predictions. Such practice hinders the progress of scientific inquiry into the factors affecting health behavior and the associated processes involved. We propose guidelines for researchers to adopt when testing the nomological validity of models applied to health behavior research to ensure appropriate interpretation when testing models and provide tests that are sufficiently robust to contribute support for, or disconfirmation of, their models. Adherence to the guidelines will enable researchers to progress the science of health behavior through rigorous tests of theories.

## Predictive and Nomological Validity

Although frequently unacknowledged, tests of social cognitive theories applied to health behavior are, in essence, tests of nomological validity. Many researchers will be familiar with multiple forms validity in the social sciences, particularly in the context of establishing the adequacy of psychometric instruments used to tap psychological constructs in the models. Tests of methodological validity include face validity (e.g., whether a set of items of a measure of a psychological construct appear to capture the essence of the construct), construct validity (e.g., whether the set of items converge on a single factor that represents the psychological construct), discriminant validity (e.g., whether the construct is distinct from other conceptually related constructs), and concurrent validity (e.g., whether the measure of the construct is correlated with other like measures of the construct). Such tests of validity are often considered prerequisites to be confirmed prior to the testing of substantive hypotheses focusing on relations between constructs and outcomes of interest, often related to behavior (Hagger, 2014). Assuming validity evidence of measures used to tap the component psychological constructs of models, researchers can then proceed to test predicted relations among the constructs according to the theory. Predictive and nomological validity are the forms of validity that pertain to the testing of proposed relations among constructs in models.

Predictive validity reflects the extent to which a single factor or variable relates to another variable of interest, either as an antecedent or consequent. For example, in the domain of health behavior, a predictive validity test might involve the prediction that a social cognitive construct predicts a behavioral outcome. Predictive validity tests should entail precise specification of the relation between the variables (e.g., directionality, valence) and boundary conditions (e.g., auxiliary assumptions) *a priori*, and be interpreted in light of any additional factors that may affect the test and the quality of the test. Within the bounds and limitations of the observation (e.g., validity evidence of the measures, the representativeness of the sample, statistical power), and based on a ‘reasonable’ falsification principle (Trafimow, 2009), the empirical test and its interpretation then represents a single datum to inform knowledge and theory on the proposed effect.

The complexity of human behavior suggests that two-variable systems, although informative, are alone insufficient in accounting for the determinants of health behavior and necessitate multifactorial, complex explanations. Testing social cognitive models in health contexts will invariably require the specification of relations among multiple constructs. Such models specify multiple relations among variables in a nomological network, and each relation in the network can be considered a single predictive validity test between two variables. Nomological validity is confirmed when all of the relations that comprise the network are supported in a single, omnibus test of model. Bagozzi (1981) notes:

“Nomological validity refers to the degree to which predictions in a formal theoretical network containing a construct of interest are confirmed. In one sense, the difference between predictive and nomological validity is one of degree and not kind. Predictive validity entails the relationship of measures of a variable to a single antecedent or consequent. Nomological validity, in contrast, involves many antecedents and/or consequents in a complex system” (p. 327).

As in the case of predictive validity, tests of the nomological validity of a model require that the specification of the pattern of effects among model variables precedes observation. Each pathway or relation in the model must therefore be specified precisely and forms part of the overall test of nomological validity. In such networks, variables can act as antecedents, consequents, or both. Furthermore, many social cognitive models applied in health and behavioral medicine contexts aim to identify the factors that determine behavior and other salient health-related outcomes (e.g., psychological well-being, quality of life), as well as the processes and mechanisms involved. Nomological networks provide the opportunity to specify patterns of relations among constructs that reflect mechanisms such as additive (multiple factors explain unique variance in outcomes), mediation (one or more factors serve to explain or *transmit* the effect of one variable on another), and moderation (one or more factors *change* the pattern of the effect of one factor on another) effects (Perugini, 2005; Michie et al., 2007; MacKinnon and Luecken, 2008; Hayes, 2013; Chatzisarantis et al., 2015). There are also opportunities for multiple combinations of these processes, such as moderated mediation (e.g., the mediated effect of one variable on another through a third variable is conditional on a moderator variable) (Wiedemann et al., 2009; Zhou et al., 2015; Hamilton and Hagger, 2017). The network will therefore comprise multiple predictions and patterns of relations among constructs in a model that reflect the researcher’s expectations as to how the behavioral phenomenon works.

Also consistent with tests of predictive validity, assessments of nomological networks must stand up to the notion of ‘reasonable’ falsifiability. As a consequence, tests should seek to validate the nomological network “as a whole” (p. 299) (Cronbach and Meehl, 1955). That is, all of the *a priori* specified predictions proposed in the network must be verified for the test to be viewed as supporting the network. Failure to find support for any one prediction within the model should raise doubts over the nomological validity of the theory represented by the network and grounds for subsequent modification that would need to be subjected to further tests.

Of course, as with tests of individual predictions, the possibility of alternative auxiliary assumptions may be able to explain the failure of some of the component predictions of the network, and, therefore, the network itself. However, such conditions should be specified *a priori*. The absence of such specification invites opportunity to explain away any test by, for example, alternative hypotheses or auxiliary assumptions, rendering the proposed network unfalsifiable. Importantly, the tests should incorporate the theory predictions and the conditions under which the predictions should hold in accordance with auxiliary assumptions. Such tests might involve, for example, testing the network under conditions determined by moderating variables which determine whether the predictions hold and reflect the auxiliary assumptions which may be candidate means to explain away failed tests of theory prediction. Such tests may provide appropriately risky tests of the network and, if they fail, may point to problems with the network rather than problems with the auxiliary assumptions (Trafimow, 2012).

## Use of Confirmatory Analytic Approaches in Nomological Validity

The advent of confirmatory analyses based on regression and covariance structures, such as path analysis and structural equation modeling, has provided researchers with powerful analytic tools by which to test simultaneously nomological frameworks specified *a priori* (Bagozzi, 2010; Hoyle, 2011). These analytic techniques enable the researcher to specify the proposed network among factors and then test the adequacy of the proposed network to explain relations among data collected on those factors. The analyses provide estimates as to whether the proposed model fits with the observations through overall evaluation of model fit and individual tests of each hypothesis that comprises the network (e.g., direct effects, mediation effects). To the extent that model fit with the data is adequate and, most importantly, support is found for each hypothesis stipulated in the network, the researcher can claim support for nomological validity. Application of these techniques in psychology has increased substantially as advanced analytic software with user-friendly interfaces has facilitated access to the complex analyses (MacCallum and Austin, 2000).

With great (statistical) power comes great responsibility, so researchers must utilize these tools appropriately when testing models applied to the health domain. Specifically, researchers must pay close attention to the specification of the model and the associated auxiliary assumptions. The analytic methods allow researchers to be extremely specific in their predictions and account for multiple predictions and associated assumptions. The closer the correspondence between the researcher’s predictions and the specification of the model to be analyzed the greater its value in contributing to evidence as a test of the theory. One of the problems associated with an over-reliance on indices indicating overall model fit using these approaches is that they can be somewhat forgiving of theories that do not quite fit the data (Marsh et al., 2004). A failure to support some of the pathways may not sufficiently compromise model fit to warrant rejection, particularly if the test focuses on evaluating difference from the null rather than a specified size and direction of the effect. Consistent with calls to focus on effect size rather than statistical significance and null hypothesis significance testing (Trafimow and Rice, 2009; Cumming, 2014; Chavalarias et al., 2016; McShane et al., 2017), researchers would do well to specify an expected effect size (e.g., a small, medium, or large effect based on Cohen’s taxonomy of effect sizes), a range of values for the effect, or the smallest effect size of interest, based on previous evidence for each prediction within the model tested (Lakens, 2014). This level of specificity increases the stringency of test of the nomological network and increases its validity as a contribution to evidence in support of, or disconfirming, the model.

## Failure to Falsify

Although most social cognitive models applied to predict health behavior and health-related outcomes in health psychology and behavioral medicine are essentially nomological networks, few researchers cite nomological validity when testing their models. In addition, although many researchers utilize the confirmatory analytic approaches reviewed previously, few adequately specify all of the predictions within their proposed network *a priori*, including relevant auxiliary assumptions, or clarify what would constitute failure to support the predictions of the theory. Instead, researchers tend to focus on testing isolated individual predictions within the network. This focus would be sufficient if testing those predictions were the sole concern. However, in many cases, researchers claim to support the predictions of the theory, even if one or more of the effects in their specified network fail to be supported by the data. Researchers tend, therefore, to adopt an heuristic of *minimal sufficiency* to evaluate their models, such that failure to find one effect among many is considered a relatively trivial ‘failure’ in the face of support for all others. Such an interpretation is inconsistent with the notion of nomological validity and presents problems for the falsifiability of model tests. If a researcher dismisses a failure to support one of the predictions within their nomological network, it presents a problem for identifying conditions under which the theory could be falsified. Of course, the researcher could attribute the failed prediction within the network to an auxiliary assumption. However, it would be important that the assumptions were specified *a priori* if the test is to make a contribution to evidence in support of, or rejecting, the network. So, opting not to reject a test of a network in the face of failed support for one or more of the *a priori* specified predictions, or specified auxiliary assumptions, potentially opens up the possibility any model could be considered acceptable. A related problem is that researchers may be tempted ‘fit hack’^1^ that is, to modify their *a priori* model though *ad hoc* inclusion or deletion of paths with the goal of finding a model that yields goodness-of-fit indices that conform to guidelines for a well-fitting model. ‘Fit hacking’ compromises nomological validity tests by capitalizing on chance to find a well-fitting model.

## Nomological Validity Tests: An Illustrative Analysis

We contend that researchers testing social cognitive models in the health behavior domain do not routinely provide explicit statements as to whether their data support or refute the nomological network of the model that is being tested. We also contend that in some cases researchers have claimed support for a model comprising multiple predictions when their test has failed to support one or more predictions, without providing adequate explanation, due to auxiliary assumptions or otherwise. To illustrate this expectation, we conducted a brief review of the research testing four key social cognitive theories in leading health psychology and behavioral medicine journals. The purpose of the research was to identify whether researchers testing the theories provide: (1) statements that they were testing the specified theory in its generic, unmodified form; (2) specification of the predictions that comprise nomological network, along with associated auxiliary assumptions; and (3) report whether or not their test provided support for, or rejection of, the hypothesized network. Our analysis is aimed at illustrating the extent to which researchers in the field provide unclear or inadequate *a priori* specification of models to be tested and how this issue affects interpretation of the test as sufficient evidence to support or disconfirm models.

### Method

We conducted a search of published correlational research testing four key social cognitive theories (the theory of reasoned action, health belief model, protection motivation theory, the theory of planned behavior) published in five key outlets in health psychology and behavioral medicine (*Annals of Behavioral Medicine, British Journal of Health Psychology*, *Health Psychology*, *Journal of Behavioral Medicine*, and *Psychology and Health*) using the *Web of Science* database. To keep the analysis manageable, we restricted our search to a 15-year period (2002–2016). Search terms and inclusion criteria are provided in Appendix A. To be eligible for inclusion, studies had to be empirical articles reporting a test of one of the four theories in its original core form in a health behavior context. Studies testing both motivational (e.g., protection motivation, intention) and behavioral outcomes were included. Studies testing hypotheses relating to additional variables within the theory or moderators of theory relations were included provided tests of the nomological validity of the theory in its core form were separable from tests of the additional hypotheses or moderators. Studies testing the theories of reasoned action and planned behavior were included if they tested the unique effects of attitudes and subjective norms, and perceived behavioral control in the case of the theory of planned behavior, on intentions in a health behavior context. Studies with a follow-up measure of behavior were included if they tested the unique effect of intentions on behavior. Studies testing the health belief model were included if they tested the unique effects of perceived severity, perceived vulnerability, perceived benefits, and perceived barriers on intentions or behavior. Studies testing protection motivation theory included if they tested unique effects of perceived vulnerability, perceived severity, self-efficacy/response efficacy, perceived response costs/barriers, and intention/protection motivation.

We coded whether researchers made an explicit statement of support or rejection of the theory according to their findings, and whether they reported findings that were inconsistent with their tests of the core hypotheses of the theory. Statements relating to qualified support or that the tests were consistent with previous research rather than the theory were not considered affirmation of support or rejection. We noted instances where researchers found effects that were not statistically significant, or explicitly labeled as trivial in size, in their tests of the sets of hypotheses that comprise the theory, which should signal failed support for the nomological network. We also coded whether researchers provided an explicitly stated null hypothesis or criteria that would lead to the failure to support hypotheses in the proposed network (e.g., finding an effect in the network considered trivial in size). Many theories specify indirect or mediated effects, and we coded whether researchers included tests of the indirect effects in their theory tests (e.g., indirect effects of appraisals on behavior through protection motivation in protection motivation theory). However, including a test of mediation was not specified as an inclusion criterion. This requirement was considered too restrictive as many of the studies focused exclusively on behavioral intention as a dependent variable and did not, therefore, test mediated effects of the belief-based constructs (e.g., attitudes, risk perceptions, norms, perceived control) on behavior. We also coded the target behavior of the studies (some studies included multiple samples and multiple behaviors), whether the key dependent variable was motivational (e.g., intention, protection motivation) or behavioral or both, and any additional variables included in augmented or modified versions of the theory tests. Full details of coding of study characteristics are provided in Appendix A (Supplementary Materials).

### Results

Studies identified in the search (*k* = 407) were screened initially for duplicates, article type (abstracts were excluded), and relevance. The remaining studies (*k* = 275) were subjected to full-text screening against eligibility criteria. The exclusion process is summarized in the flow diagram in Appendix B (Supplementary Materials). A full list of studies included the final analysis (*k* = 122) and studies excluded (*k* = 153) with reasons for exclusion are provided in Appendices C and D (Supplementary Materials), respectively. Characteristics of included studies with key data for analysis are provided in Appendix E (Supplementary Materials). Ten studies tested more than one theory leaving the total number of theory tests at 133.

Of the 133 theory tests, 87 (65.41%) stated that they were testing the theory of interest in its core form, whereas others did not claim to test the theory or presented hypotheses relating to alternative variables or augmented versions of the theory. Only 39 tests (29.32%) made an explicit statement in support of (*k* = 32; 24.06%), or rejecting (*k* = 7; 5.26%), the hypotheses of the theory or model, while the remainder (*k* = 94; 70.68%) did not include a statement claiming to support or reject theory predictions on the basis of their data. Among those claiming to test a theory (*k* = 87), 50 (57.47%) did not provide an explicit statement of support for theory predictions. A substantial number of theory tests (*k* = 84; 63.15%) reported at least one finding that was contrary to theory hypotheses. Prominent examples of effects that were frequently found to be contrary to theory predictions were effects of perceived severity and susceptibility on intentions or protection motivation in protection motivation theory, and effects of subjective norms and perceived behavioral control on intentions in the theory of planned behavior. Of those claiming to test a theory (*k* = 87; 65.41%), 60 (68.97%) reported at least one finding contrary to theory predictions. We were particularly interested in claims for theory support among researchers claiming to test the theory when they reported at least one finding contrary to its predictions. Among researchers claiming to test the theory (*k* = 87), 15 (17.24%) claimed support for the theory when their data suggested that at least one prediction within the network was contrary to hypotheses, whereas 15 (17.24%) claimed support when their data suggested acceptance, and 7 (8.05%) indicated that the predictions of the theory should be rejected when their data indicated that the theory predictions should be rejected. Of the substantial proportion of theory tests (*k* = 50; 57.47%) that did not make a claim of support or rejection, 38 (76.00%) reported at least one finding contrary to hypotheses. None of the studies stated a null hypothesis or reported data that would lead to the rejection of the theory tested. Furthermore, none of the studies included hypotheses that made explicit reference to effect size. Finally, only 17 theory tests (14.66%) tested indirect effects in their analysis.

### Discussion

Our analysis illustrates that the majority of authors of included studies did not make an explicit statement either supporting or rejecting the predictions of the theory of interest, and this finding was the case regardless of whether researchers claimed to test the theory of interest in its original form or not. This brief review illustrates an important point when it comes the testing social cognitive theories in health contexts: explicit statements supporting the predictions of the theory being tested are not routinely provided. In addition, a substantial number of researchers reporting findings contrary to the predictions of the theory being tested do not make explicit statements rejecting those predictions, and, in some cases, claimed support for theory predictions when the data indicated otherwise. From the perspective of testing for nomological validity, current findings suggest that researchers, and, by implication, those making decisions on published research, do not make their claims for support or rejection of the nomological networks they test explicit. Exclusion of this detail makes it difficult to ascertain the researcher’s position as to whether the network of relations among theory variables being tested is acceptable or should be rejected and subsequently revised consistent with the nomological validity approach. In place of explicit statements of support or rejection, researchers frequently fall back on qualified statements which are uninformative regarding the acceptability of the *a priori* specified network, or is akin to *a posteriori* hypothesizing without *a priori* specification of the conditions that would lead to the rejection of the theory (e.g., auxiliary assumptions). For example, researchers frequently provide qualified statements claiming “partial support” for the network or that their findings that “are largely consistent with” previous research, and we have not been immune to making such statements (Hagger et al., 2009; Barkoukis and Hagger, 2013). Similarly, researchers frequently make reference to previous research that reported similar findings that were contrary to hypotheses to justify their failure to find the same effect, or cite methodological limitations or sample-specific idiosyncrasies as explanations. Surprisingly, few entertain the notion that the network should be rejected.

The current analysis illustrates the imperative of identifying the criteria necessary for a nomological validity test to provide support for the propositions of a theory. The researcher should, therefore, identify the ‘core’ theory components and the associated set of relations among the components that are the minimum required for the test to support, or fail to support, the theory. Judgments also need to be made as to which components, and the relations among them, are central or ‘core’ to the theory, and which should be conditional on auxiliary assumptions. For example, effects of attitudes, subjective norms, and perceived behavioral control on intentions, and intentions on behavior, comprise key hypotheses of the theory of planned behavior. While these hypotheses may be considered ‘core’ to the theory, the relative strength or size of the effect of each component is expected to vary across context. For example, the effect of subjective norms on intentions is expected to be stronger in groups that endorse collectivist values, or for co-operative behaviors like blood donation, and smaller or even zero in groups endorsing individualist values or behaviors that are highly personal like exercising alone. As a consequence, finding a trivial or null effect for subjective norms on intention in a nomological test of the theory would not lead to a conclusion that the propositions of the theory should be rejected. However, it would be imperative to specify the auxiliary assumptions on which the subjective norm-intention relationship is conditional in advance, rather than hypothesizing after the fact. Auxiliary assumptions may apply to other relations in the model, and they should be subject to auxiliary assumptions specified *a priori*. It is also important that the hypotheses conditional on the auxiliary assumptions are tested systematically in subsequent tests of the theory. Some hypotheses within nomological tests may be flagged as ‘core’ to the theory. For example, in the theory of planned behavior, the effect of intentions on behavior is considered fundamental (McEachan et al., 2012; Rich et al., 2015). While such effects may also be subject to auxiliary assumptions, such assumptions may affect the strength but not the presence of the effect. Hypotheses relating to fundamental effects should, therefore, be stated consistent with this prediction. Current findings indicate that researchers do no routinely pay close consideration to identifying ‘core’ effects in nomological validity tests of theories, or specify auxiliary assumptions affecting theory effects *a priori*. Such a practice does not lead to strong evidence to support or reject a theory, and may lead to *post hoc* explanations when findings do not conform to expectations.

### Limitations and Conclusion

It is important to note the limitations of the current analysis. We based our judgements on the presence or absence of theory effects tested in the studies included in the current analysis based on the available evidence including statistical significance, effect size, and the interpretation presented by the authors. However, this approach does not rule out the possibility that that decisions regarding the presence of effects may have been affected by a lack of statistical power or methodological limitations in the studies. Related to this, none of the articles specified the size of the component effects or pathways that comprised the theories tested. Where predictions were specified, they were almost exclusively in terms of presence or absence of the effect, and relied on null hypothesis significance tests. As research evidence testing networks of relations in a social cognitive theory expands, specification of effects with greater precision is possible and necessary to advance knowledge on the true pattern of theory effects, an issue we will return to later in the section on Bayesian approaches to nomological validity. It is also important to note that the sample of studies included in our analysis did not encompass all research on social cognitive models in health behavior and was restricted to a relatively narrow 15-year period. The limited breadth of our literature search was commensurate with the illustrative purpose of our analysis and we believe it provides a reasonably accurate depiction of theory-driven research adopting social cognitive theories in health behavior contexts. Finally, the current analysis was conducted on research adopting correlational designs, which have inherent limitations with respect to inference of causality. However, our approach could also apply to experimental or intervention studies in which one (or more) variables within the nomological network was manipulated and effects of the manipulations on other constructs in the model tested. The network could be tested by including the manipulated variables as dummy-coded variables alongside measures of other model constructs in a path analysis or structural equation model. In conclusion, our analysis illustrates a pervading problem in research testing theories in health behavior contexts: researchers claiming to test theories tend not to state *a priori* the hypothesized network of relations among theory constructs and associated auxiliary assumptions; tend not to state *a priori* the conditions that will lead to the tested network being rejected, and tend not to provide explicit statement of support or rejection of the predictions of the network based on their findings.

## Fundamental vs. Exploratory Pathways in Nomological Networks

Although the strict criteria for nomological validity advocated by Cronbach and Meehl (1955) provides a framework for the falsifiability of models, it is recognized as overly restrictive, not least because failures to support the predictions of a network could be attributed to the network or to auxiliary assumptions. Absolute falsifiability is, therefore, unrealistic. However, nomological networks that make risky predictions along with diligent specification of auxiliary assumptions make the potential of ‘reasonable’ falsification possible. But what about exploratory tests? Sometimes researchers have no *a priori* specification of the existence or direction of particular predictions in a model. Such exploratory tests can be incorporated within tests of a theory, but should not be specified as part of the nomological network, and, therefore, should not be involved in the decision to accept or reject the model based on a test of its nomological validity. Importantly, exploratory pathways should also be identified *a priori* in the same way that confirmatory pathways fundamental to the nomological validity tests are specified prior to observation. In doing so, researchers are able to clearly lay out the criteria used to determine the validity of a nomological network in subsequent tests.

Hagger and Chatzisarantis (2016b) suggest that researchers make the distinction between fundamental and exploratory or peripheral effects when specifying relations among variables in a nomological network. Fundamental effects reflect the hypothesized relations that comprise the formal nomological network. Empirical support for all of the fundamental relations is required in tests for nomological validity, taking into account any specified auxiliary assumptions. In contrast, exploratory or peripheral effects are those that are non-essential for the nomological network as the theory or conceptual basis of the effect has not been resolved, perhaps due to competing hypotheses or lack of prior knowledge. Such effects may be of interest theoretically and testing them may inform future theorizing and serve as the basis for revised models, but they serve no role in determining the acceptance or rejection of the proposed model. Both fundamental and exploratory pathways are identified, along with any auxiliary assumptions, *a priori* consistent with the nomological validity approach. Thus, when it comes to the interpretation of empirical tests of nomological validity, it is clear which effects in the network should be taken into account when making the decision as to whether the model should be accepted or rejected, and which effects should be disregarded when making that decision. This approach enables the researcher to test a proposed nomological network, but simultaneously test exploratory hypotheses that may be of theoretical importance, but not relevant to the proposed model. As Hagger and Chatzisarantis stress, the *a priori* specification of all paths in a network is a condition of this approach, and posteriori formulation of the model or re-designation of paths as exploratory is to be avoided.

## Meta-Analytic Structural Equation Modeling and Nomological Validity

Evaluation of evidential support for the predictions of a nomological network or its failure should be evaluated in the context of the quality of the tests on which the evidence is based. Limitations inherent in empirical studies testing nomological networks may limit the extent to which the study stands as sufficient in providing strong evidence for or against the predictions of the network. For example, studies may be confined to a narrow group that is insufficiently representative of the population, or use methods that inadequately capture constructs of interest or are subject to bias that introduces variability unattributed to the effects in the tested network. Such methodological artifacts of sampling and measurement error provide caveats as to whether tests are sufficient to make decision on the adequacy of a nomological network. The limitations in the conduct of empirical tests may cast doubt on any decision on whether the network should be accepted or rejected. As a consequence, single tests of a theory should be interpreted in light of the adequacy of the methods used, the appropriateness of the sample, and auxiliary assumptions known or assumed to affect relations in the network.

There is also potential for chance findings to provide misleading tests of nomological validity. Researchers have reported experiencing difficulty in replicating some of the most influential effects in social psychology (Open Science Collaboration, 2012; Earp and Trafimow, 2015; Hagger et al., 2016b). Such findings have been attributed to the tendency for journal editors to favor research that provides support for novel effects, even in cases where the study may be underpowered and the size of effects found are disproportionate in relation to the sample sizes on which they were tested (Pashler and Harris, 2012). Such large effects in small underpowered studies suggest that the findings may have occurred due to chance and that many more probably null findings are rejected for publication or suppressed by authors who do not think they could be published, or, worse still, do not want them to be published (Hagger and Chatzisarantis, 2014). As a solution, researchers have advocated the importance of the replication of findings using identical methods and in samples that were appropriately powered (Ritchie et al., 2012; Zwaan, 2014). In the context of nomological validity, multiple replications of model tests in large, representative samples are advocated to minimize the potential for false positive tests of hypotheses within nomological networks occurring due to chance.

Given the potential for study quality and chance findings to affect conclusions in tests of nomological validity, we advocate that confirmatory support for theories in the health domain and beyond should be based on converging evidence through multiple, high-powered replications of nomological validity tests. The tests should adopt valid methods that are fit-for-purpose in tapping the required constructs and minimizing sampling and measurement error. This means that failure to support the nomological network should be interpreted on the context of the precision and quality of the data on which the failed test is reliant. A single failed test of a network is unlikely to be considered sufficient to abandon a theory, particularly if questions can be raised over the integrity or validity of the data, but cumulative evidence with multiple high-quality failed replications raises uncertainty over the nomological validity of the theory.

When a sufficient body of evidence is available, the replications should also be subjected to meta-analytic path analysis or structural equation modeling, which provides a powerful means to evaluate the cumulative evidence for the nomological network while correcting for methodological inadequacies such as measurement and sampling error (Hagger et al., 2016a; Cheung and Hong, 2017). A two-stage approach is advocated in which tests of each relation between constructs involved in the nomological network are corrected for sampling error using meta-analysis, and then the model of interest is tested using the meta-analytically corrected matrix of relations among the variables. The resultant model represents a test of nomological validity based on the cumulative evidence from multiple replications. It represents a robust test in that it is derived from multiple replications and is corrected for artifacts that may have led to a researcher making an incorrect decision as to the acceptance or rejection of the network. The adoption of this analytic approach is on the rise (e.g., Yu et al., 2007; Carraro and Gaudreau, 2013; Hagger et al., 2016a, 2017; Cheung and Hong, 2017; Credé et al., 2017; Protogerou et al., 2017). For example, Carraro and Gaudreau (2013) conducted a meta-analytic path analysis testing a nomological network in which variables representing planning for physical activity (action planning and coping planning) mediated the relation between intentions and physical activity participation. However, relatively few studies that have adopted the omnibus testing of theory-based nomological networks based on cumulative data from meta-analyses. We advocate that researchers adopt such an approach in future tests of models in health psychology.

## A Bayesian Approach to Testing A Nomological Network

A limitation of the traditional meta-analytic path analysis approach to research synthesis is that it requires the availability of a sufficient body of work before one can quantitatively assess the cumulative evidence for a nomological network. Although a meta-analysis can be performed with two primary studies, statistical power to detect moderate-sized effects in the network often falls well below 80% when there is fewer than six primary studies (Borenstein et al., 2009). Depending on the scholarly interest in a topic, it may take years for tests of nomological validity to accumulate and permit a retrospective meta-analysis. Of course, there may be instances in which a meta-analysis may be planned prospectively whereby a consortium of researchers work together in replicating a nomological network with the intention of statistical synthesis through meta-analysis (Eerland et al., 2016; Hagger et al., 2016b). Nevertheless, large-scale projects that involve multiple researchers across several labs are time consuming and costly, and therefore represent the exception rather than the rule when it comes to accumulation of knowledge.

It is often the case that researchers conduct a study against the backdrop of previous theory or research, yet prior knowledge of effects in a theory is typically ignored in frequentist statistics that rely on statistical significance testing, such that scholars test “the same null hypothesis over and over again” (van de Schoot et al., 2014) (p. 843). An alternative approach is offered by Bayesian estimation, which is growing in use as an analytic method in psychology (van de Schoot et al., 2017). Bayesian estimation is not reliant on the existence of a sufficient body of evidence for statistical synthesis of data testing a theory or a model. In Bayesian analysis an existing theory or evidence serves as a starting point to inform the analysis and is updated with new data (Muthén and Asparouhov, 2012; Zyphur and Oswald, 2015). Prior knowledge can come from a variety of sources, including theoretical expectations, expert knowledge, or evidence from pilot data, individual studies or meta-analytic estimates (van de Schoot et al., 2014; Zyphur and Oswald, 2015). When testing a new theory, for example, one can formally incorporate theoretical predictions regarding the direction (e.g., effect is positive and therefore ranges between 0 and 1) and magnitude (e.g., low, moderate, large effect) of the proposed network of relations among theory constructs into the analyses, consistent with the nomological validity approach.

From a Bayesian perspective, prior information can be thought of being situated on a continuum ranging from substantial uncertainty (non-informative) to a great deal of certainty (informative) in one’s expectations about the nature of effects or relations in a theory (Depaoli et al., 2017). The level of (un)certainty with respect to each hypothesized effect in a theoretical network is quantified in a probability distribution, known as a ‘prior,’ which considers a range of plausible values rather than a specific value for each effect (van de Schoot et al., 2014; Zyphur and Oswald, 2015). For example, guided by meta-analytic data, one might expect the highest plausibility for a standardized effect of variable *X* on variable *Y* in a nomological network to center on 0.40, with values below 0.10 or above 0.70 considered highly unlikely (i.e., 95% credibility interval). This prior distribution is combined with new data via Bayes’ theorem to produce the posterior distribution for the effect that represents an updated summary of what is known about the effect (Muthén and Muthén, 2012), akin to an ‘automatic’ meta-analysis (Zyphur and Oswald, 2015). Continuing with the previous example, if the combination of prior knowledge with new data indicates that the effect of *X* on *Y* is centered on 0.30 (95% credibility interval = 0.14, 0.46), one may conclude that there is a 95% likelihood that the true effect ranges between 0.14 and 0.46. This intuitive interpretation of the 95% interval in Bayesian estimation differs from that of the frequentist statistics, where it represents the 95% probability that other unobserved intervals obtained through repeated sampling would contain the true population parameter (Hoekstra et al., 2014). Whereas the frequentist approach is focused on the probability of the data, given a set of assumptions captured in a statistical model, Bayesian estimation is concerned with the probability that the predictions of the theory are true, given the data (Zyphur and Oswald, 2015). Thus, Bayesian estimation permits inferences to be made about effects within a theoretical network, which is what many researchers want to know.

In terms of nomological validity, Bayesian estimation facilitates tests of the validity of a network of relations of a theory as well as individual relations (Zyphur and Oswald, 2015). Through posterior predictive checking (Muthén and Muthén, 2012; Gelman et al., 2013), Bayesian estimation makes falsification possible via an assessment of the fit of a model, representing the network of relations in a theory, to a specific dataset and an understanding of those aspects of the data that are incongruent with the model (Gelman et al., 2013). Posterior predictive checking involves a comparison of the observed versus generated data and samples of the parameters from the posterior distribution; taking into consideration both of these sources of uncertainty is what provides Bayesian analysis with an advantage over frequentist approaches (Zyphur and Oswald, 2015). To quantify model fit, the posterior predictive *p*-value provides an indication of the proportion of times the posterior distribution – obtained from the combination of prior beliefs and new data – resembles the observed data (van de Schoot et al., 2014; Zyphur and Oswald, 2015). Values around 0.50 indicate a well-fitting model, whereas small values (e.g., *p <* 0.05) suggest poor model-data fit because the generated data are more probable than the observed data (Muthén and Muthén, 2012; Zyphur and Oswald, 2015).

In addition to model fit, examinations of the posterior distributions permits inferences that are intuitive and relevant for falsification of nomological validity. When testing a new theory in the absence of empirical data, for example, one can directly test and make inferences regarding theoretical expectations concerning the direction (i.e., positive or inverse) of the relations among theory constructs. For a positive effect of a specific relation within a theory, a researcher might examine the proportion of the *a posteriori* specified distribution that falls between standardized values of 0.20 and 1 (or whatever range is considered meaningful within the context of the theory); if a large proportion existed in this credibility interval, it would provide support for the expectation of a meaningful effect. For example, Gucciardi and Jackson (2015) conducted a two-wave longitudinal study to examine a theoretical sequence in which basic psychological needs satisfaction from peer and adults leaders (e.g., coach) determined athletes’ attitudes, subjective norms, and perceived behavioral control and, in turn, intentions to remain involved in sport. Perceived behavioral and intentions served as the primary determinants of sport continuation approximately 12 months later. Both empirical (i.e., meta-analytic data on the relations among the theory of planned behavior constructs) and theoretical information (i.e., moderate positive association between basic psychological needs and the social-cognitive variables of the theory of planned behavior) guided the development of Bayesian priors that were integrated with new data collected from a sample of young adults. In summary, Bayesian approaches offer an advance on frequentist approaches when testing the nomological validity of health behavior theories by providing the opportunity to test theories based on prior information including theory and data. Theories can therefore be specified *a priori* based on its predictions and available evidence, subjected to subsequent testing, verified or rejected, and modified and updated.

## Guidelines for Model Testing Based on Nomological Validity

We advocate that researchers consider nomological validity when testing the adequacy of social cognitive models aimed at predicting health behavior and health-related outcomes. We expect that testing for nomological validity involving networks of relations among theory variables will encourage researchers to specify clearly the predictions required for the theory to be supported, along with associated auxiliary assumptions, and prevent selective *post hoc* justification for failed tests and ‘fit hacking.’ We provide a set of four guidelines for researchers adopting the nomological approach: (1) Specification. Clear specification of the proposed effects or hypotheses in a model consistent with the nomological approach, including the any auxiliary assumptions on which the predictions are conditional; (2) Investigation. Identify appropriate tests that allow confirmation or rejection of the network specified *a priori* against observations or data; (3) Interpretation. Make a definitive decision based on the empirical test without making *post hoc* adjustments or judgements that go against the *a priori* specification of the network and auxiliary assumptions; and (4) Replication or reformulation. Use the interpretation in (3) as a basis for further confirmatory replications to ensure the network is robust and replicable or, pending a decision to reject the network, formal respecification of the model based on theory and the previous test and subject it to further validation on a fresh body of data (Cronbach and Meehl, 1955). We provide details of these four guidelines in the next sections.

(1) Specification. Clear *a priori* specification of the relations among proposed constructs of a model based on priori theory and research is essential for a nomological validity test. The researcher needs to specify clearly the directional pathways in the network among constructs in the model, as well as hypothesized effects relating to processes, including direct, indirect, and reciprocal relations, and potential moderation effects. In addition, if any effects are not considered fundamental to the test of the model, then they should be clearly identified *a priori* as exploratory or peripheral. Such a position has been advocated by numerous researchers aiming to promote better model specification (Sniehotta et al., 2014; Hagger and Chatzisarantis, 2016b). It is also important that the specification of the fundamental effects in the model are, wherever possible, accompanied by statements of effect size estimates or a range of potential effect sizes. This information will prevent acceptance of models when one or more of the *a priori* specified pathways are so small that they are considered trivial from a practical or theoretical perspective, even when tests of such pathways surpass criteria for statistical significance. Finally, clear specification of the auxiliary assumptions expected to affect predictions (e.g., moderator variables that might magnify, diminish, or extinguish proposed effects) is required. A useful means to aid the clarification of the specification of a nomological network is to plot a path diagram in which constructs and pathways are represented by ‘boxes’ and ‘arrows.’ Useful resources exist on effective means to draw diagrams to accurately capture the pathways and processes of interest. We encourage researchers to study the conceptual diagrams proposed by Hayes’ (2013), which provide multiple hypothetical examples to illustrate patterns of effects in nomological networks. These diagrams not only have the advantage of guiding the researcher on how to depict the proposed network, but also directly relate to the potential analytic procedures that might be adopted to test the network in empirical data sets.
(2) Investigation. Researchers are encouraged to adopt rigorous research designs (e.g., longitudinal or experimental designs), use measures with sound psychometric integrity, collect data on suitable samples with adequate statistical power, and adopt appropriate confirmatory analytic techniques when testing nomological networks. Maximizing the quality of evidence to support a network should be a guiding principle. At this stage the researcher should also specify the expected size of the effects in the nomological network and base the selection of the sample size on those effects. Tools to estimate sample size and statistical power for complex path analytic models with indirect effects using Monte Carlo simulations have recently been made available (Schoemann et al., 2017). Consistent with the requirement for sound data and recent advocacy for strong data to test effects in social psychology (Open Science Collaboration, 2015), researchers should also aim for stringent alpha levels and high >0.90 statistical power to control for type I and type II error rates, respectively. Researchers should also ensure the adoption of measures with sufficient reliability and validity to tap constructs of interest in order to minimize measurement error in tests of model hypotheses. In the cases of cumulative evidence testing nomological networks across existing data sets through path analytic meta-analysis, researchers should clearly specify inclusion criteria and adopt rigorous consensus methods to ensure equivalence of measures across studies included in the synthesis. The researcher should also be mindful of potential heterogeneity in effect sizes across studies and to search for possible moderators of effects within the network.
(3) Interpretation. Tests for nomological validity demand that the entire network is tested as a whole, and failure of individual predictions means failed support for nomological validity of the model. Researchers should avoid temptations to claim support for the model if a proposed prediction fundamental to the network is not supported. Of course, this interpretation must be made with due consideration of potential mitigating factors such as auxiliary assumptions and data quality based on a reasonable falsification criterion (Trafimow, 2012). Posteriori justification of tests that fail to support proposed networks undermine scientific progress by making model development an unstructured, subjective process that lies outside the bounds of scientific rigor. Trends toward the pre-registration of study hypotheses, protocols, and analytic procedures is a useful means to restrict *post hoc* decision making (Probst and Hagger, 2015; Jonas and Cesario, 2016).
(4) Replication or reformulation. If researchers interpret their test as supportive of the nomological validity of their proposed model, then they should seek to exactly or conceptually replicate the effects, perhaps considering varying extraneous conditions relating to auxiliary assumptions (Trafimow, 2009). Even if the researcher has been diligent in conducting the research using measures with sound reliability and validity evidence, and in an appropriately powered sample that is reasonably representative of the population of interest, a single supportive test of nomological validity is insufficient to provide definitive support. In order to verify the effects and to ensure that the initial test was not obtained by chance, replication is advocated (Lindsay, 2015). Over time cumulative failures to support a model may catalyze researchers to specify a revised nomological network. The network should be treated as a ‘new’ model and subjected to new tests of nomological validity consistent with steps 1 to 3.

These recommendations place the onus on researchers to adopt a stringent nomological approach when it comes to testing models. Adopting them will limit researchers introducing alternative hypotheses after the fact to explain findings that run contrary to hypotheses and avoid falsification evasion. Of course, those responsible for determining the direction of published science and the promulgation of findings in journals also have a significant role to play. Journal editors and peer reviewers are also well placed to demand that research submitted for publication adopts rigorous tests of nomological validity and provides clear demarcation of the conditions that lead to the falsification of predictions. Similarly, professors and teachers have a responsibility to advocate the nomological approach to ensure that students are versed in the principles of reasonable falsifiability as they embark on their fledgling research careers.

## Conclusion

Testing the adequacy of psychological models to explain health-related behavior and outcomes provides an evidence base for future theory-based behavioral interventions (Glanz and Bishop, 2010; Hagger et al., 2012; Gourlan et al., 2016). In this article we have discussed the merits of rigorous nomological validity tests of models applied in health psychology. We have argued that such an approach is important to generate strong supportive evidence for models and minimize *ad hoc* posteriori explanations for failed predictions. We argue that current means to test models tend not to adopt this strict approach and do not provide a strong basis on which to decide whether a network has been supported or falsified. We have provided a set of guidelines for researchers to promote more effective, fit-for-purpose model tests using the nomological approach. We also identified the importance of converging evidence for the nomological validity of models across multiple tests through path analytic meta-analysis and the role of Bayesian estimation to provide greater precision tests of nomological validity based on prior evidence. Finally, while we have illustrated the imperative of adopting rigorous nomological validity tests when applying social cognitive theories in health behavior research, a domain in which understanding of the antecedents and processes that lead to behavior is a priority, such tests should also be advocated in other behavioral domains in which social cognitive theories have been applied. Our suggested guidelines for nomological validity tests should also be adopted in tests of social cognitive theories applied in educational, environmental, and prosocial behavioral domains. We encourage researchers and journal editors to assume responsibility in ensuring that tests of social cognitive theories in multiple applied disciplines are subjected to strong tests of nomological validity.

## Author Contributions

MH conceived the ideas presented in the manuscript with assistance from DG and NC. MH, DG, and NC drafted the manuscript.

## Conflict of Interest Statement

The authors declare that the research was conducted in the absence of any commercial or financial relationships that could be construed as a potential conflict of interest.

## Footnotes

1 We thank one of the reviewers for suggesting this term.
