# Supplementary material for: On Nomological Validity and Auxiliary Assumptions: The Importance of Simultaneously Testing Effects in Social Cognitive Theories Applied to Health Behavior and Some Guidelines
Source: Front Psychol. 2017 Nov 3;8:1933. doi: 10.3389/fpsyg.2017.01933 (PMC5675876; doi:10.3389/fpsyg.2017.01933)
Supplement: Supplementary file 1 [file Appendix_A_Strategy_and_Inclusion_Criteria.pdf]

Appendix A: Search Terms, Inclusion Criteria and Coding Details for Studies in Illustrative Analysis

Search Terms

Theory of Reasoned Action

Year range: 2002-2016

Search terms: Topic: “theory of reasoned action”; Publication Name: “Health Psychology” or “Journal of Behavioral Medicine” or “Annals of Behavioral Medicine” or “British Journal of Health Psychology” or “Psychology & Health”

Number of items: 14

Health Belief Model

Year range: 2002-2016

Search terms: Topic: “health belief model”; Publication Name: “Health Psychology” or “Journal of Behavioral Medicine” or “Annals of Behavioral Medicine” or “British Journal of Health Psychology” or “Psychology & Health”

Number of items: 56

Protection Motivation Theory

Year range: 2002-2016

Search terms: Topic: “Protection motivation theory”; Publication Name: “Health Psychology” or “Journal of Behavioral Medicine” or “Annals of Behavioral Medicine” or “British Journal of Health Psychology” or “Psychology & Health”

Number of items: 55

Theory of Planned Behaviour

Year range: 2002-2016

Search terms: Topic: “theory of planned behav*”; Publication Name: “Health Psychology” or “Journal of Behavioral Medicine” or “Annals of Behavioral Medicine” or “British Journal of Health Psychology” or “Psychology & Health”

Number of items: 282

Inclusion Criteria for Tests of Theories

Theory of Reasoned Action. Research testing the theory of reasoned action had to measure and test unique effects of attitudes and subjective norms on intentions in a health behaviour context. Studies including follow-up behavioral measures had to test the unique

effect of intentions on behaviour. Study constructs could be measured using direct measures or indirect, belief-based measures.

Theory of planned behavior. Research testing the theory of planned behavior had to measure and test unique effects of attitudes, subjective norms, and perceived behavioral control on intentions in a health behaviour context. Studies including follow-up behavioral measures had to test the unique effect of intentions on behaviour. Study constructs could be measured using direct measures or indirect, belief-based measures.

Health belief model. Research testing the health belief model had to test the unique effects of perceived severity, perceived vulnerability, perceived benefits, and perceived barriers on intentions or behaviour in a health behaviour context.

Protection motivation theory. Research testing the health belief model had to test the unique effects of perceived vulnerability, perceived severity, self-efficacy/response efficacy, perceived response costs/barriers, and intention/protection motivation in a health behaviour context.

Study coding

Detailed criteria adopted to code studies included in the analysis in the following is presented in the following sections corresponding to the column headings in Table E1, Appendix E.

Claim to test theory. Researchers making reference to testing the theory using terms such as testing the "ability" or "efficacy" of the theory to explain an outcome, without being qualified as an augmented, extended or modified version of the theory, it was considered an explicit that they aimed to test the hypotheses of theory, that is, its nomological network.

Claim for support or rejection of the model. References to support for the theory including references to "support", "confirmation", "corroboration", "sufficiency", "utility", "successful", and "validity" of the theory, or references to "in line with previous research" or "results are to a large extent in agreement with the theory", were interpreted as claims for support provided they made clear reference to the theory itself and not ancillary hypotheses or hypotheses relating to augmented versions of the theory. Terms referring to the "rejection", "contradiction", "disconfirmation", "failure" were interpreted as a lack of support and rejection of the theory. Statements suggesting that the theory test was consistent with previous applications of the theory or previous analyses were not considered statements of support or rejection. Statements of qualified support (e.g., "most of the theory predictions held"; the theory was "partially successful"; "mixed support was found…") were not considered sufficient as statement supporting the test of the theory.

Hypothesized pathway(s) found to be contrary to hypotheses. Tests of effects from the hypothesized theoretical network of the core theory that were not found to be statistically significant effects, or effect labelled as trivial, were identified and listed in full including the any sample-specific information.

Falsification hypothesis. Researcher-specified null hypothesis or criteria that would lead to a rejection of the a priori specified network for the core theory test was identified. A "no" response reflected the absence of a null hypothesis or rejection criteria.

Tested indirect effects. We also indicated whether the researchers tested indirect effects proposed in the models to illustrate the extent to which researchers had tested these hypotheses, but we considered this too restrictive as an inclusion criterion.

Behavior, **dependent variable(s)**, and **additional variable(s)/augmented version of theory**. We also coded the target behaviour(s) of the studies (some studies included multiple samples and multiple behaviors), whether the key dependent variable was motivational (e.g., intention, protection motivation) or behavioral or both, and any additional variables included in augmented or modified versions of the theory tests. Research testing hypotheses relating to additional variables within the theory or moderators of the theory relations was included provided tests of the nomological validity of theory in its core form were discernible from tests of the additional hypotheses. Studies were therefore required to report tests of the core theory hypotheses in the absence of the additional constructs.
