# Supplementary figures and images for: On Nomological Validity and Auxiliary Assumptions: The Importance of Simultaneously Testing Effects in Social Cognitive Theories Applied to Health Behavior and Some Guidelines
Source: Front Psychol. 2017 Nov 3;8:1933. doi: 10.3389/fpsyg.2017.01933 (PMC5675876; doi:10.3389/fpsyg.2017.01933)

## Appendix B. Flow Diagram for Studies Included in Illustrative Analysis

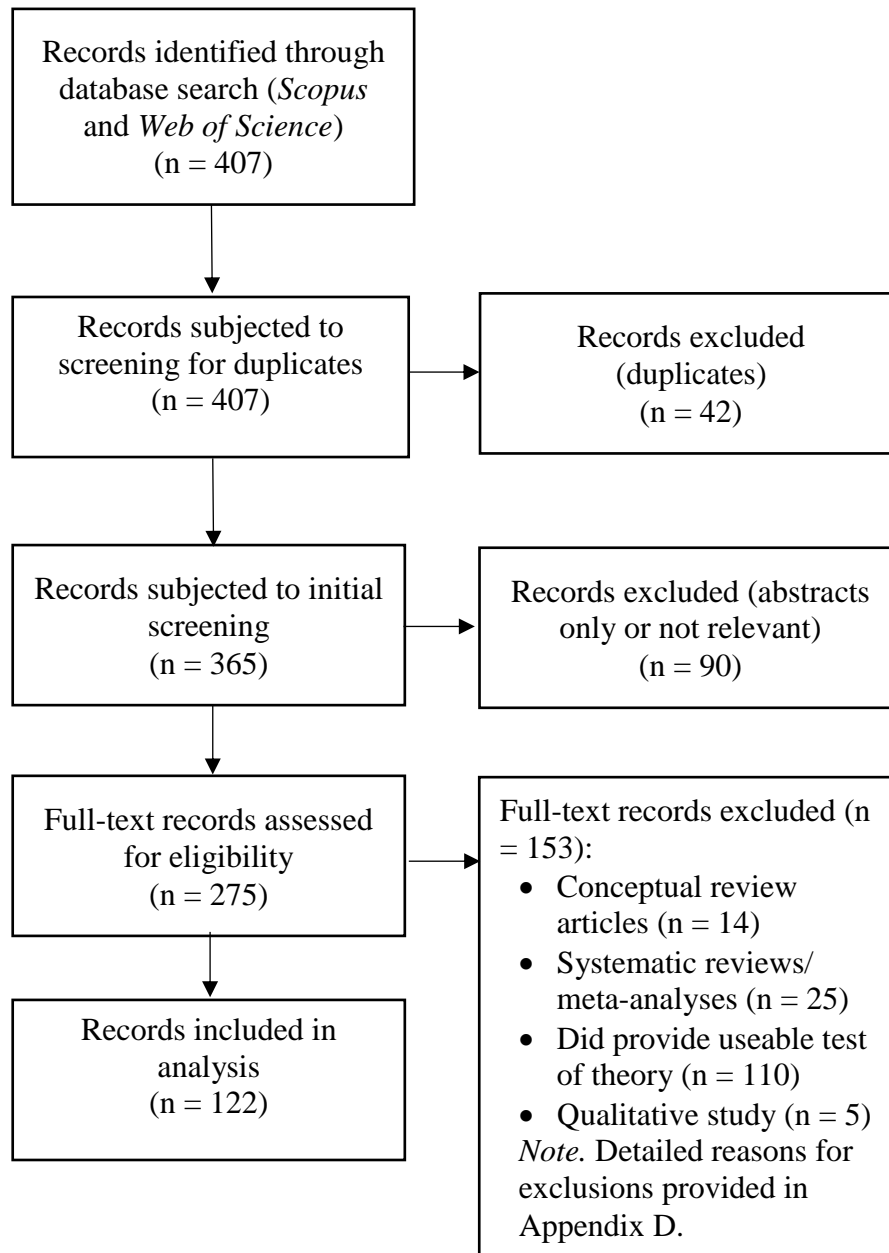

Supplement: Supplementary file 2 [file Appendix_B_Flow_Diagram.pdf]
