# Supplementary material for: On Nomological Validity and Auxiliary Assumptions: The Importance of Simultaneously Testing Effects in Social Cognitive Theories Applied to Health Behavior and Some Guidelines
Source: Front Psychol. 2017 Nov 3;8:1933. doi: 10.3389/fpsyg.2017.01933 (PMC5675876; doi:10.3389/fpsyg.2017.01933)
Supplement: Supplementary file 3 [file Appendix_C_Articles_included.pdf]

Appendix A: Articles Included in Illustrative Analysis

Abraham, C., Henderson, M., & Der, G. (2004). Cognitive impact of a research-based school sex education programme. *Psychology & Health*, *19*(6), 689–703. doi: 10.1080/08870440410001722921

Abraham, C. & Sheeran, P. (2004). Deciding to exercise: The role of anticipated regret. *British Journal of Health Psychology, 9*(2), 269–278. doi: 10.1348/135910704773891096

Araujo-Soares, V., Rodrigues, A., Presseau, J., & Sniehotta, F. F. (2013). Adolescent sunscreen use in springtime: A prospective predictive study informed by a belief elicitation investigation. *Journal of Behavioral Medicine*, *36*(2), 109–123. doi: 10.1007/s10865-012-9415-3

Arbour-Nicitopoulos, K. P., Ginis, K. A. M., & Wilson., P. M. (2010). Examining the individual and perceived neighborhood associations of leisure-time physical activity in persons with spinal cord injury. *Annals of Behavioral Medicine*, *39*(2), 192–197. doi: 10.1007/s12160-009-9149-9

Armitage, C. J. (2005). Can the Theory of Planned Behaviour Predict the Maintenance of Physical Activity? *Health Psychology*, *24*(3), 235–245. doi: 10.1037/0278-6133.24.3.235

Armitage, C. J., Norman, P., & Conner, M. (2002). Can the Theory of Planned Behaviour mediate the effects of age, gender and multidimensional health locus of control? *British Journal of Health Psychology*, *7*, 299–316. doi: 10.1348/135910702760213698

Bagot, K. L., Masser, B. M., & White, K. M. (2015). Using an Extended Theory of Planned Behavior to Predict a Change in the Type of Blood Product Donated. *Annals of Behavioral Medicine*, 510–521. doi: 10.1007/s12160-014-9677-9

Boer, H., & Mashamba, M. T. (2007). Gender power imbalance and differential psychosocial correlates of intended condom use among male and female adolescents from Venda, South Africa. *British Journal of Health Psychology*, *12*, 51–63. doi: 10.1348/135910706X102104

Bonetti, D., Johnston, M., Clarkson, J., & Turner, S. (2009). Applying multiple models to predict clinicians' behavioural intention and objective behaviour when managing children's teeth. *Psychology & Health*, *24*(7), 843–60. doi: 10.1080/08870440802108918

Booth, A. R., Norman, P., Harris, P. R., & Goyder, E. (2014). Using the theory of planned behaviour and self-identity to explain chlamydia testing intentions in young people living in deprived areas. *British Journal of Health Psychology*, *19*(1), 101–112. doi: 10.1111/bjhp.12036

Braithwaite, D., Sutton, S., & Steggles, N. (2002). Intention to Participate in Predictive Genetic Testing for Hereditary Cancer: The Role of Attitude toward Uncertainty. *Psychology & Health*, *17*(6), 761–772. doi: 10.1080/0887044021000054764

Brickell, T. a, Chatzisarantis, N. L. D., & Pretty, G. M. (2006). Using past behaviour and spontaneous implementation intentions to enhance the utility of the theory of planned behaviour in predicting exercise. *British Journal of Health Psychology, 11*(2), 249–262. doi: 10.1348/135910705X52471

Browne, J. L., & Chan, a Y. C. (2012). Using the Theory of Planned Behaviour and implementation intentions to predict and facilitate upward family communication about mammography. *Psychology & Health*, *27*(6), 655–73. doi: 10.1080/08870446.2011.615396

Bryan, A., Fisher, J. D., & Fisher, W. A. (2002). Tests of the mediational role of preparatory safer sexual behavior in the context of the theory of planned behavior. *Health*

Psychology, 21(1), 71–80. doi: 10.1037//0278-6133.21.1.71
Byrne, C., Walsh, J., Kola, S., & Sarma, K. M. (2012). Predicting intention to uptake H1N1 influenza vaccine in a university sample. *British Journal of Health Psychology, 17*(3), 582–595. doi: 10.1111/j.2044-8287.2011.02057.x
Ch'Ng, J. W. M., & Glendon, A. I. (2014). Predicting sun protection behaviors using protection motivation variables. *Journal of Behavioral Medicine, 37*(2), 245–256. doi: 10.1007/s10865-012-9482-5
Chan, D. K. C., Fung, Y. K., Xing, S., & Hagger, M. S. (2014). Myopia prevention, near work, and visual acuity of college students: Integrating the theory of planned behavior and self-determination theory. *Journal of Behavioral Medicine, 37*(3), 369–380. doi: 10.1007/s10865-013-9494-9
Chan, D. K.-C., Yang, S. X., Mullan, B., Du, X., Zhang, X., Chatzisarantis, N. L. D., & Hagger, M. S. (2015). Preventing the spread of H1N1 influenza infection during a pandemic: Autonomy-supportive advice versus controlling instruction. *Journal of Behavioral Medicine, 38*(3), 416–426. doi: 10.1007/s10865-014-9616-z
Chatzisarantis, N. L. D., & Hagger, M. S. (2008). Influences of personality traits and continuation intentions on physical activity participation within the theory of planned behaviour. *Psychology & Health, 23*(3), 347–367. doi: 10.1080/14768320601185866
Churchill, S., & Jessop, D. C. (2011). Reflective and non-reflective antecedents of health-related behaviour: exploring the relative contributions of impulsivity and implicit self-control to the prediction of dietary behaviour. *British Journal of Health Psychology, 16*(2), 257–272. doi: 10.1348/135910710X498688
Conner, M., & Godin, G. (2007). Temporal stability of behavioural intention as a moderator of intention–health behaviour relationships. *Psychology & Health, 22*(8), 875–897. doi: 10.1080/14768320601070449
Conner, M., Godin, G., Sheeran, P., & Germain, M. (2013). Some Feelings Are More Important: Cognitive Attitudes, Affective Attitudes, Anticipated Affect, and Blood Donation. *Health Psychology, 32*(3), 264–72. doi: 10.1037/a0028500
Conner, M., McEachan, R., Taylor, N., O'Hara, J., & Lawton, R. (2015). Role of affective attitudes and anticipated affective reactions in predicting health behaviors. *Health Psychology, 34*(6), 642–652. doi: 10.1037/hea0000143
Conner, M., Norman, P., & Bell, R. (2002). The theory of planned behavior and healthy eating. *Health Psychology, 21*(2), 194–201. doi: 10.1037//0278-6133.21.2.194
Conner, M., Sandberg, T., McMillan, B., & Higgins, A. (2006). Role of anticipated regret, intentions and intention stability in adolescent smoking initiation. *British Journal of Health Psychology, 11*(1), 85–101. doi: 10.1348/135910705X40997
Conner, M., Sandberg, T., & Norman, P. (2010). Using action planning to promote exercise behavior. *Annals of Behavioral Medicine, 40*(1), 65–76. doi: 10.1007/s12160-010-9190-8
Conner, M., Sutherland, E., Kennedy, F., Grearly, C., & Berry, C. (2008). Impact of alcohol on sexual decision making: Intentions to have unprotected sex. *Psychology & Health, 23*(8), 909–934. doi: 10.1080/08870440701596551
Cooke, R., & French, D. P. (2011). The role of context and timeframe in moderating relationships within the theory of planned behaviour. *Psychology & Health, 26*(9), 1225–1240. doi: 10.1080/08870446.2011.572260
Courneya, K. S., Conner, M., & Rhodes, R. E. (2006). Effects of different measurement scales on the variability and predictive validity of the "two-component" model of the theory of planned behavior in the exercise domain. *Psychology & Health, 21*(5), 557–570. doi: 10.1080/14768320500422857
Courneya, K. S., Friedenreich, C. M., Sela, R. a, Quinney, H. A., & Rhodes, R. E. (2002).

Correlates of adherence and contamination in a randomized controlled trial of exercise in cancer survivors: an application of the theory of planned behavior and the five factor model of personality. *Annals of Behavioral Medicine 24*(4), 257–268. doi: 10.1207/S15324796ABM2404_02

de Bruijn, G.-J., Brug, J., & Van Lenthe, F. J. (2009). Neuroticism, conscientiousness and fruit consumption: exploring mediator and moderator effects in the theory of planned behaviour. *Psychology & Health*, *24*(9), 1051–1069. doi: 10.1080/08870440802428241

De Bruijn, G.-J., Kremers, S. P. J., De Vet, E., De Nooijer, J., Van Mechelen, W., & Brug, J. (2007). Does habit strength moderate the intention–behaviour relationship in the Theory of Planned Behaviour? The case of fruit consumption. *Psychology & Health*, *22*(8), 899–916. doi: 10.1080/14768320601176113

Drossaert, C. H. C., Boer, H., & Seydel, E. R. (2003). Prospective study on the determinants of repeat attendance and attendance patterns in breast cancer screening using the theory of planned behaviour. *Psychology & Health*, *18*(5), 551–565. doi: 10.1080/0887044031000141207

Farquharson, L., Noble, L. M., Barker, C., & Behrens, R. H. (2004). Health beliefs and communication in the travel clinic consultation as predictors of adherence to malaria chemoprophylaxis. *British Journal of Health Psychology*, *9*(2), 201–217. doi: 10.1348/135910704773891050

Gardner, B., De Bruijn, G. J., & Lally, P. (2012). Habit, identity, and repetitive action: A prospective study of binge-drinking in UK students. *British Journal of Health Psychology*, *17*(3), 565–581. doi: 10.1111/j.2044-8287.2011.02056.x

Gerend, M. A., & Shepherd, J. E. (2012). Predicting human papillomavirus vaccine uptake in young adult women: Comparing the health belief model and theory of planned behavior. *Annals of Behavioral Medicine*, *44*(2), 171–180. doi: 10.1007/s12160-012-9366-5

Gorin, S. S. (2005). Correlates of colorectal cancer screening compliance among urban hispanics. *Journal of Behavioral Medicine*, *28*(2), 125–137. doi: 10.1007/s10865-005-3662-5

Gredig, D., Nideroest, S., & Parpan-Blaser, A. (2007). Explaining the condom use of heterosexual men in a high-income country: Adding somatic culture to the theory of planned behaviour. *Journal of Public Health*, *15*(2), 129–140. doi: 10.1007/s10389-007-0091-7

Hagger, M. S., Chatzisarantis, N. L. D., & Biddle, S. J. H. (2002). The influence of autonomous and controlling motives on physical activity intentions within the Theory of Planned Behaviour. *British Journal of Health Psychology*, *7*(3), 283–297. doi: 10.1348/135910702760213689

Hagger, M. S., Chatzisarantis, N. L. D., Hein, V., Soos, I., Karsai, I., Lintunen, T., & Leemans, S. (2009). Teacher, peer and parent autonomy support in physical education and leisure-time physical activity: A trans-contextual model of motivation in four nations. *Psychology & Health*, *24*(6), 689–711. doi: 10.1080/08870440801956192

Hagger, M. S., Lonsdale, A. J., Hein, V., Koka, A., Lintunen, T., Pasi, H., … Chatzisarantis, N. L. D. (2012). Predicting alcohol consumption and binge drinking in company employees: An application of planned behaviour and self-determination theories. *British Journal of Health Psychology*, *17*(2), 379–407. doi: 10.1111/j.2044-8287.2011.02043.x

Hanbury, A., Wallace, L., & Clark, M. (2009). Use of a time series design to test effectiveness of a theory-based intervention targeting adherence of health professionals to a clinical guideline. *British Journal of Health Psychology*, *14*(3), 505–518. doi: 10.1348/135910708X369558

Hardeman, W., Kinmonth, A. L., Michie, S., & Sutton, S. (2011). Theory of planned behaviour cognitions do not predict self-reported or objective physical activity levels or

change in the ProActive trial. *British Journal of Health Psychology*, *16*(1), 135–150. doi: 10.1348/135910710X523481

Hassandra, M., Vlachopoulos, S. P., Kosmidou, E., Hatzigeorgiadis, A., Goudas, M., & Theodorakis, Y. (2011). Predicting students' intention to smoke by theory of planned behaviour variables and parental influences across school grade levels. *Psychology & Health*, *26*, 1241–1258. doi: 10.1080/08870446.2011.605137

Hay, J. L., Ford, J. S., Klein, D., Primavera, L. H., Buckley, T. R., Stein, T. R., … Ostroff, J. S. (2003). Adherence to colorectal cancer screening in mammography-adherent older women. *Journal of Behavioral Medicine*, *26*(6), 553–576. doi: 10.1023/A:1026253802962

Hukkelberg, S. S., Hagtvet, K. A., & Kovac, V. B. (2014). Latent interaction effects in the theory of planned behaviour applied to quitting smoking. *British Journal of Health Psychology*, *19*(1), 83–100. doi: 10.1111/bjhp.12034

Hunter, M. S., Grunfeld, E. A., & Ramirez, A. J. (2003). Help-seeking intentions for breast-cancer symptoms: a comparison of the self-regulation model and the theory of planned behaviour. *British Journal of Health Psychology*, *8*(3), 319–33. doi: 10.1348/135910703322370888

Huston, S. a, Bagozzi, R. P., & Kirking, D. M. (2010). Decision-making about the use of hormone therapy among perimenopausal women. *British Journal of Health Psychology*, *15*(2), 231–51. doi: 10.1348/135910709X457946

Hyde, M. K., & White, K. M. (2009). Communication prompts donation: exploring the beliefs underlying registration and discussion of the organ donation decision. *British Journal of Health Psychology*, *14*(3), 423–35. doi: 10.1348/135910708X339542

Hyde, M. K., & White, K. M. (2010). Are organ donation communication decisions reasoned or reactive? A test of the utility of an augmented theory of planned behaviour with the prototype/willingness model. *British Journal of Health Psychology*, *15*(2), 435–452. doi: 10.1348/135910709X468232

Inauen, J., Tobias, R., & Mosler, H. J. (2014). The role of commitment strength in enhancing safe water consumption: Mediation analysis of a cluster-randomized trial. *British Journal of Health Psychology*, *19*(4), 701–719. doi: 10.1111/bjhp.12068

Jellema, I. J., Abraham, C., Schaalma, H. P., Gebhardt, W. A., & Van Empelen, P. (2013). Predicting having condoms available among adolescents: The role of personal norm and enjoyment. *British Journal of Health Psychology*, *18*(2), 453–468. doi: 10.1111/j.2044-8287.2012.02088.x

Johnston, K. L., & White, K. M. (2003). Binge-drinking: A test of the role of group norms in the theory of planned behaviour. *Psychology & Health*, *18*(1), 63–77. doi: Doi 10.1080/0887044021000037835

Jones, C. J., Smith, H. E., Frew, A. J., Toit, G. Du, Mukhopadhyay, S., & Llewellyn, C. D. (2014). Explaining adherence to self-care behaviours amongst adolescents with food allergy: A comparison of the health belief model and the common sense self-regulation model. *British Journal of Health Psychology*, *19*(1), 65–82. doi: 10.1111/bjhp.12033

Jones, L. W., Courneya, K. S., Fairey, A. S., & Mackey, J. R. (2005). Does the theory of planned behavior mediate the effects of an oncologist's recommendation to exercise in newly diagnosed breast cancer survivors? Results from a randomized controlled trial. *Health Psychology*, *24*(2), 189–97. doi: 10.1037/0278-6133.24.2.189

Kassem, N. O., & Lee, J. W. (2004). Understanding soft drink consumption among male adolescents using the theory of planned behavior. *Journal of Behavioral Medicine*, *27*(3), 273–296. doi: 10.1023/B:JOBM.0000028499.29501.8f

Kiviniemi, M. T., Voss-Humke, A. M., & Seifert, A. L. (2007). How do I feel about the behavior? The interplay of affective associations with behaviors and cognitive beliefs as

influences on physical activity behavior. *Health Psychology*, *26*(2), 152–158. doi: 10.1037/0278-6133.26.2.152

Kor, K., & Mullan, B. A. (2011). Sleep hygiene behaviours: An application of the theory of planned behaviour and the investigation of perceived autonomy support, past behaviour and response inhibition. *Psychology & Health*, *26*(9), 1208–1224. doi: 10.1080/08870446.2010.551210

Kothe, E. J., & Mullan, B. A. (2014). Interaction effects in the theory of planned behaviour: Predicting fruit and vegetable consumption in three prospective cohorts. *British Journal of Health Psychology*, *20*(3), 549–562. doi: 10.1111/bjhp.12115

Krawczyk, A. L., Perez, S., Lau, E., Holcroft, C. a, Amsel, R., Knäuper, B., & Rosberger, Z. (2012). Human papillomavirus vaccination intentions and uptake in college women. *Health Psychology*, *31*(5), 685–93. doi: 10.1037/a0027012

Latimer, A. E., & Martin Ginis, K. a. (2005). The importance of subjective norms for people who care what others think of them. *Psychology & Health*, *20*(1), 53–62. doi: 10.1080/08870440412331300002

Lawton, R., Ashley, L., Dawson, S., Waiblinger, D., & Conner, M. (2012). Employing an extended Theory of Planned Behaviour to predict breastfeeding intention, initiation, and maintenance in White British and South-Asian mothers living in Bradford. *British Journal of Health Psychology*, *17*(4), 854–871. doi: 10.1111/j.2044-8287.2012.02083.x

Legare, F., Godin, G., Dodin, S., Turcot, L., & Laperriere, L. (2003). Adherence to hormone replacement therapy: A longitudinal study using the theory of planned behaviour. *Psychology & Health*, *18*(3), 351–371. doi: 10.1080/0887044031000146824

Manne, S., Markowitz, A., Winawer, S., Meropol, N. J., Haller, D., Rakowski, W., … Jandorf, L. (2002). Correlates of colorectal cancer screening compliance and stage of adoption among siblings of individuals with early onset colorectal cancer. *Health Psychol*, *21*(1), 3–15. doi: 10.1037//0278-6133.21.1.3

Matterne, U., Diepgen, T. L., & Weisshaar, E. (2011). A longitudinal application of three health behaviour models in the context of skin protection behaviour in individuals with occupational skin disease. *Psychology & Health*, *26*, 1188–1207. doi: 10.1080/08870446.2010.546859

McMillan, B., Conner, M., Green, J., Dyson, L., Renfrew, M., & Woolridge, M. (2009). Using an extended theory of planned behaviour to inform interventions aimed at increasing breastfeeding uptake in primiparas experiencing material deprivation. *British Journal of Health Psychology*, *14*(2), 379–403. doi: 10.1348/135910708X336112

McMillan, B., Conner, M., Woolridge, M., Dyson, L., Green, J. M., Renfrew, M. J., … Clarke, G. (2008). Predicting breastfeeding in women living in areas of economic hardship: explanatory role of the theory of planned behaviour. *Psychology & Health*, *23*(7), 767–88. doi: 10.1080/08870440701615260

Mercken, L., Candel, M., van Osch, L., & de Vries, H. (2011). No smoke without fire: The impact of future friends on adolescent smoking behaviour. *British Journal of Health Psychology*, *16*(1), 170–88. doi: 10.1348/135910710X531608

Michie, S., Dormandy, E., French, D. P., & Marteau, T. M. (2004). Using the theory of planned behaviour to predict screening uptake in two contexts. *Psychology & Health*, *19*(6), 705–718. doi: 10.1080/08870440410001704930

Milne, S., Orbell, S., & Sheeran, P. (2002). Combining motivational and volitional interventions to promote exercise participation: Protection motivation theory and implementation intentions. *British Journal of Health Psychology*, *7*, 163–184. doi: 10.1348/135910702169420

Milton, A. C., & Mullan, B. A. (2012). An application of the theory of planned behavior—a randomized controlled food safety pilot intervention for young adults. *Health*

Psychology, 31(2), 250–259. doi: 10.1037/a0025852
Moan, I. S., & Rise, J. (2011). Predicting intentions not to "drink and drive" using an extended version of the theory of planned behaviour. *Accident Analysis and Prevention, 43*(4), 1378–1384. doi: 10.1016/j.aap.2011.02.012
Moan, I. S., Rise, J., & Andersen, M. (2005). Predicting parents' intentions not to smoke indoors in the presence of their children using an extended version of the theory of planned behaviour. *Psychology & Health, 20*, 353-371. doi:10.1080/08870440512331317706
Montanaro, E. A., & Bryan, A. D. (2014). Comparing theory-based condom interventions: Health belief model versus theory of planned behavior. *Health Psychology, 33*(10), 1251–1260. doi: 10.1037/a0033969
Morrison, D. M., Lohr, M. J., Beadnell, B. A., Gillmore, M. R., Lewis, S., & Gilchrist, L. (2010). Young mothers' decisions to use marijuana: a test of an expanded Theory of Planned Behaviour. *Psychology & Health*, *25*(5), 569–587. doi: 10.1080/08870440902777554
Moser, S. E., & Aiken, L. S. (2011). Cognitive and emotional factors associated with elective breast augmentation among young women. *Psychology & Health*, *26*(1), 41–60. doi: 10.1080/08870440903207635
Murnaghan, D. A, Blanchard, C. M., Rodgers, W. M., LaRosa, J. N., MacQuarrie, C. R., MacLellan, D. L., & Gray, B. J. (2010). Predictors of physical activity, healthy eating and being smoke-free in teens: a theory of planned behaviour approach. *Psychology & Health*, *25*(8), 925–941. doi: 10.1080/08870440902866894
Murphy, C. C., Vernon, S. W., Diamond, P. M., & Tiro, J. A. (2014). Competitive testing of health behavior theories: How do benefits, barriers, subjective norm, and intention influence mammography behavior? *Annals of Behavioral Medicine*, *47*(1), 120–129. doi: 10.1007/s12160-013-9528-0
Newton, J. D., Newton, F. J., Ewing, M. T., Burney, S., & Hay, M. (2013). Conceptual overlap between moral norms and anticipated regret in the prediction of intention: implications for theory of planned behaviour research. *Psychology & Health*, *28*(5), 495–513. doi: 10.1080/08870446.2012.745936
Norman, P., & Brain, K. (2005). An application of an extended health belief model to the prediction of breast self-examination among women with a family history of breast cancer. *British Journal of Health Psychology*, *10*(1), 1–16. doi: 10.1348/135910704X24752
Norman, P., & Conner, M. (2006). The theory of planned behaviour and binge drinking: Assessing the moderating role of past behaviour within the theory of planned behaviour. *British Journal of Health Psychology*, *11*(1), 55–70. doi: 10.1348/135910705X43741
Norman, P., & Cooper, Y. (2011). The theory of planned behaviour and breast self-examination: Assessing the impact of past behaviour, context stability and habit strength. *Psychology & Health, 26*, 1156-1172. doi:10.1080/08870446.2010.481718
Norman, P., Searle, A., Harrad, R., & Vedhara, K. (2003). Predicting adherence to eye patching in children with amblyopia: An application of protection motivation theory. *British Journal of Health Psychology*, *8*(1), 67–82. doi: 10.1348/135910703762879219
Orbell, S., & Hagger, M. (2006a). Temporal framing and the decision to take part in type 2 diabetes screening: effects of individual differences in consideration of future consequences on persuasion. *Health Psychology*, *25*(4), 537–548. doi: 10.1037/0278-6133.25.4.537
Orbell, S., & Hagger, M. (2006b). "When no means no": can reactance augment the theory of planned behavior? *Health Psychology*, *25*(5), 586–594. doi: 10.1037/0278-6133.25.5.586

Orbell, S., Hagger, M., Brown, V., & Tidy, J. (2006). Comparing two theories of health behavior: a prospective study of noncompletion of treatment following cervical cancer screening. *Health Psychology*, *25*(5), 604–15. doi: 10.1037/0278-6133.25.5.604

Orbell, S., Lidierth, P., Henderson, C. J., Geeraert, N., Uller, C., Uskul, A. K., & Kyriakaki, M. (2009). Social-cognitive beliefs, alcohol, and tobacco use: a prospective community study of change following a ban on smoking in public places. *Health Psychology*, *28*(6), 753–61. doi: 10.1037/a0016943

Payne, N., Jones, F., & Harris, P. R. (2004). The role of perceived need within the theory of planned behaviour: A comparison of exercise and healthy eating. *British Journal of Health Psychology*, *9*(4), 489–504. doi: 10.1348/1359107042304524

Payne, N., Jones, F., & Harris, P. R. (2005). The impact of job strain on the predictive validity of the theory of planned behaviour: an investigation of exercise and healthy eating. *British Journal of Health Psychology*, *10*, 115–131. doi: 10.1348/135910704X14636

Plotnikoff, R. C., Lippke, S., Courneya, K., Birkett, N., & Sigal, R. (2010). Physical activity and diabetes: An application of the theory of planned behaviour to explain physical activity for Type 1 and Type 2 diabetes in an adult population sample. *Psychology and Health*, *25*(1), 7–23. doi: 10.1080/08870440802160984

Plotnikoff, R. C., Lippke, S., Trinh, L., Courneya, K. S., Birkett, N., & Sigal, R. J. (2010). Protection motivation theory and the prediction of physical activity among adults with type 1 or type 2 diabetes in a large population sample. *British Journal of Health Psychology*, *15*(3), 643–661. doi: 10.1348/135910709X478826

Plotnikoff, R. C., Lubans, D. R., Penfold, C. M., & Courneya, K. S. (2014). Testing the utility of three social-cognitive models for predicting objective and self-report physical activity in adults with type 2 diabetes. *British Journal of Health Psychology*, *19*(2), 329–346. doi: 10.1111/bjhp.12085

Prati, G., Mazzoni, D., & Zani, B. (2014). Perceived behavioural control, subjective norms, attitudes and intention to use condom: a longitudinal cross-lagged design. *Psychology & Health*, *29*(10), 1119–36. doi: 10.1080/08870446.2014.913043

Presseau, J., Sniehotta, F. F., Francis, J. J., & Gebhardt, W. a. (2010). With a little help from my goals: integrating intergoal facilitation with the theory of planned behaviour to predict physical activity. *British Journal of Health Psychology*, *15*(4), 905–919. doi: 10.1348/135910710X494105

Rhodes, R. E., & Blanchard, C. M. (2008). Do sedentary motives adversely affect physical activity? Adding cross-behavioural cognitions to the theory of planned behaviour. *Psychology & Health*, *23*(7), 789–805. doi: 10.1080/08870440701421578

Rhodes, R. E., Blanchard, C. M., Benoit, C., Levy-Milne, R., Naylor, P., Downs, D. S., & Warburton, D. E. R. (2014). Social cognitive correlates of physical activity across 12 months in cohort samples of couples without children, expecting their first child, and expecting their second social cognitive correlates of physical activity across 12 months. *Health Psychology*, *33*(8), 792–802. doi: 10.1037/a0033755

Rhodes, R. E., Blanchard, C. M., & Matheson, D. H. (2006). A multicomponent model of the theory of planned behaviour. *British Journal of Health Psychology*, *11*(1), 119–137. doi: 10.1348/135910705X52633

Rise, J., Kovac, V., Kraft, P., & Moan, I. S. (2008). Predicting the intention to quit smoking and quitting behaviour: extending the theory of planned behaviour. *British Journal of Health Psychology*, *13*, 291–310. doi: 10.1348/135910707X187245

Rivis, A., & Sheeran, P. (2003). Social influences and the Theory of Planned Behaviour: evidence for a direct relationship between prototypes and young people's exercise behaviour. *Psychology & Health*, *18*(5), 567–583. doi: 10.1080/0887044032000069883

Sainsbury, K., Mullan, B., & Sharpe, L. (2015). Predicting intention and behaviour following participation in a theory-based intervention to improve gluten free diet adherence in coeliac disease. *Psychology & Health*, *30*(9), 1063-1074. doi: 10.1080/08870446.2015.1022548

Schmiege, S. J., Aiken, L. S., Sander, J. L., & Gerend, M. a. (2007). Osteoporosis prevention among young women: psychosocial models of calcium consumption and weight-bearing exercise. *Health Psychology*, *26*(5), 577–87. doi: 10.1037/0278-6133.26.5.577

Scott, E. J., Eves, F. F., French, D. P., & Hoppé, R. (2007). The theory of planned behaviour predicts self-reports of walking, but does not predict step count. *British Journal of Health Psychology*, *12*(4), 601–620. doi: 10.1348/135910706X160335

Sieverding, M., Matterne, U., & Ciccarello, L. (2010). What role do social norms play in the context of men's cancer screening intention and behavior? Application of an extended theory of planned behavior. *Health Psychology*, *29*(1), 72–81. doi: 10.1037/a0016941

Smerecnik, C., Quaak, M., van Schayck, C. P., van Schooten, F.-J., & de Vries, H. (2011). Are smokers interested in genetic testing for smoking addiction? A socio-cognitive approach. *Psychology & Health*, *26*(8), 1099–112. doi: 10.1080/08870446.2010.541909

Steadman, L., & Rutter, D. R. (2004). Belief importance and the theory of planned behaviour: comparing modal and ranked modal beliefs in predicting attendance at breast screening. *British Journal of Health Psychology*, *9*(4), 447–63. doi: 10.1348/1359107042304579

Steadman, L., Rutter, D. R., & Field, S. (2002). Individually elicited versus modal normative beliefs in predicting attendance at breast screening: Examining the role of belief salience in the Theory of Planned Behaviour. *British Journal of Health Psychology*, *7*(Part 3), 317–330. doi: 10.1348/135910702760213706

Symons Downs, D., & Hausenblas, H. A. (2007). Pregnant women's third trimester exercise behaviors, body mass index, and pregnancy outcomes. *Psychology & Health*, *22*(5), 545–559. doi: 10.1080/14768320701372018

Taylor, S. D., Bagozzi, R. P., & Gaither, C. A. (2005). Decision making and effort in the self-regulation of hypertension: testing two competing theories. *British Journal of Health Psychology*, *10*(4), 505–30. doi: 10.1348/135910704X22376

Tessier, D., Sarrazin, P., Nicaise, V., & Dupont, J.-P. (2015). The effects of persuasive communication and planning on intentions to be more physically active and on physical activity behaviour among low-active adolescents. *Psychology & Health*, *30*(5), 583–604. doi: 10.1080/08870446.2014.996564

Tulloch, H., Reida, R., D'Angeloa, M. S., Plotnikoff, R. C., Morrina, L., Beatona, L., … Pipe, A. (2009). Predicting short and long-term exercise intentions and behaviour in patients with coronary artery disease: A test of protection motivation theory. *Psychology & Health*, *24*(3), 255–69. doi: 10.1080/08870440701805390

Umeh, K. (2004). Cognitive appraisals, maladaptive coping, and past behaviour in protection motivation. *Psychology & Health*, *19*(6), 719–735. doi: 10.1080/0887044042000196692

Umeh, K., & Patel, R. (2004). Theory of planned behaviour and ecstasy use: an analysis of moderator-interactions. *British Journal of Health Psychology*, *9*(1), 25–38. doi: 10.1348/135910704322778704

Vallance, J. K. H., Courneya, K. S., Plotnikoff, R. C., & Mackey, J. R. (2008). Analyzing theoretical mechanisms of physical activity behavior change in breast cancer survivors: Results from the Activity Promotion (ACTION) Trial. *Annals of Behavioral Medicine*, *35*(2), 150–158. doi: 10.1007/s12160-008-9019-x

Van den Putte, B., Yzer, M., Willemsen, M. C., & de Bruijn, G.-J. (2009). The effects of smoking self-identity and quitting self-identity on attempts to quit smoking. *Health Psychology*, *28*(5), 535–44. doi: 10.1037/a0015199

Van de Ven, M. O. M., Engels, R. C. M. E., Otten, R., & Van den Eijnden, R. J. J. M. (2007).

A longitudinal test of the theory of planned behavior predicting smoking onset among asthmatic and non-asthmatic adolescents. *Journal of Behavioral Medicine*, *30*(5), 435–445. doi: 10.1007/s10865-007-9119-2

Van Lettow, B., De Vries, H., Burdorf, A., Conner, M., & Van Empelen, P. (2015). Explaining young adults' drinking behaviour within an augmented theory of planned behaviour: Temporal stability of drinker prototypes. *British Journal of Health Psychology*, *20*(2), 305–323. doi: 10.1111/bjhp.12101

Walsh, J. J., da Fonseca, R. S., & Banta, A. (2005). Watching and participating in exercise videos: A test of the theory of planned behaviour, conscientiousness, and the role of implementation intentions. *Psychology & Health*, *20*(6), 729–741. doi: 10.1080/1476832050018786

Wang, C., Gonzalez, R., Janz, N. K., Milliron, K. J. & Merajver, S. D.. (2007). The role of cognitive appraisal and worry in BRCA1/2 testing decisions among a clinic population. *Psychology & Health*, *22*(6), 719–736 18p. doi: 10.1080/14768320600976216

White, K. M., Robinson, N. G., Young, R. M., Anderson, P. J., Hyde, M. K., Greenbank, S., … Baskerville, D. (2008). Testing an extended theory of planned behaviour to predict young people's sun safety in a high risk area. *British Journal of Health Psychology*, *13*(3), 435–448. doi: 10.1348/135910707X210004

White, K. M., Starfelt, L. C., Young, R. M., Hawkes, A. L., Leske, S., & Hamilton, K. (2015). Predicting Australian adults' sun-safe behaviour: Examining the role of personal and social norms. *British Journal of Health Psychology*, *20*(2), 396–412. doi: 10.1111/bjhp.12108

Whitford, H. M., & Jones, M. (2011). An exploration of the motivation of pregnant women to perform pelvic floor exercises using the revised theory of planned behaviour. *British Journal of Health Psychology*, *16*(4), 761–778. doi: 10.1111/j.2044-8287.2010.02013.x

Wilkinson, D., & Abraham, C. (2004). Constructing an integrated model of the antecedents of adolescent smoking. *British Journal of Health Psychology*, *9*(3), 315–333. doi: 10.1348/1359107041557075

Wolff, K., Nordin, K., Brun, W., Berglund, G., & Kvale, G. (2011). Affective and cognitive attitudes, uncertainty avoidance and intention to obtain genetic testing: An extension of the Theory of Planned Behaviour. *Psychology & Health*, *26*(9), 1143–1155. doi: 10.1080/08870441003763253

Wong, C. L., & Mullan, B. A. (2009). Predicting breakfast consumption: an application of the theory of planned behaviour and the investigation of past behaviour and executive function. *British Journal of Health Psychology, 14,* 489–504. doi: 10.1348/135910708X360719

Zhang, J., Jemmott III, J. B., & Jemmott, L. S. (2015). Mediation and moderation of an efficacious theory-based abstinence-only intervention for African American adolescents. *Health Psychology, 34*(12), 1175–1184. doi: 10.1037/hea0000244

Zimmermann, F., & Sieverding, M. (2010). Young adults' social drinking as explained by an augmented theory of planned behaviour: the roles of prototypes, willingness, and gender. *British Journal of Health*
