# Supplementary material for: On Nomological Validity and Auxiliary Assumptions: The Importance of Simultaneously Testing Effects in Social Cognitive Theories Applied to Health Behavior and Some Guidelines
Source: Front Psychol. 2017 Nov 3;8:1933. doi: 10.3389/fpsyg.2017.01933 (PMC5675876; doi:10.3389/fpsyg.2017.01933)
Supplement: Supplementary file 4 [file Appendix_D_Articles_excluded_and_Reasons.pdf]

Appendix B: Articles Excluded from Illustrative Analysis and Reasons for Exclusion

Excluded Articles

Abraham, C., Southby, L., Quandte, S., Krahé, B., & Sluijs, W. van der. (2007). What's in a leaflet? Identifying research-based persuasive messages in European alcohol-education leaflets. *Psychology & Health*, *22*(1), 31–60. doi: 10.1080/14768320600774405

Ajzen, I. (2011). The theory of planned behaviour: Reactions and reflections. *Psychology & Health*, *26*(9), 1113–1127. doi: 10.1080/08870446.2011.613995

Albarracín, D., McNatt, P. S., Klein, C. T. F., Ho, R. M., Mitchell, A. L., & Kumkale, G. T. (2003). Persuasive communications to change actions: An analysis of behavioral and cognitive impact in HIV prevention. *Health Psychology*, *22*(2), 166–177. doi: 10.1037/0278-6133.22.2.166

Alexander, A. B., Stupiansky, N. W., Ott, M. A., Herbenick, D., Reece, M., & Zimet, G. D. (2014). What parents and their adolescent sons suggest for male HPV vaccine messaging. *Health Psychology* , *33*(5), 448–56. doi: 10.1037/a0033863

Allom, V., & Mullan, B. (2012). Self-regulation versus habit: The influence of self-schema on fruit and vegetable consumption. *Psychology & Health*, *27*(sup2), 7–24. doi: 10.1080/08870446.2011.605138

Arden, M. A., & Armitage, C. J. (2008). Predicting and explaining transtheoretical model stage transitions in relation to condom-carrying behaviour. *British Journal of Health Psychology, 13*(4), 719–735. doi: 10.1348/135910707X249589

Armitage, C. J. (2004). Evidence that implementation intentions reduce fat intake: A randomized trial. *Health Psychology*, *23*(3), 319–323. doi: 10.1037/0278-6133.23.3.319

Armitage, C. J. (2008). A volitional help sheet to encourage smoking cessation: a randomized exploratory trial. *Health Psychology* , *27*(5), 557–566. doi: 10.1037/0278-6133.27.5.557

Armitage, C. J., Povey, R., & Arden, M. A. (2003). Evidence for discontinuity patterns across the stages of change: A role for attitudinal ambivalence. *Psychology & Health*, *18*(3), 373–386. doi: 10.1080/0887044031000066553

Armitage, C. J., & Arden, M. A. (2002). Exploring discontinuity patterns in the transtheoretical model : An application of the theory of planned behaviour. *British Journal of Health Psychology, 7*, 89–103. doi: 10.1348/135910702169385

Armitage, C. J., & Arden, M. A. (2008). How useful are the stages of change for targeting interventions? Randomized test of a brief intervention to reduce smoking. *Health Psychology, 27*(6), 789–798. doi: 10.1037/0278-6133.27.6.789

Armitage, C. J., & Reidy, J. G. (2008). Use of mental simulations to change theory of planned behaviour variables. *British Journal of Health Psychology, 13*(3), 513–524. doi: 10.1348/135910707X227088

Arroll, M. A., & Howard, A. (2013). "The letting go, the building up, [and] the gradual process of rebuilding": identity change and post-traumatic growth in myalgic encephalomyelitis/chronic fatigue syndrome. *Psychology & Health*, *28*(3), 302–18. doi: 10.1080/08870446.2012.721882

Atkinson, T. M., Salz, T., Touza, K. K., Li, Y., & Hay, J. L. (2015). Does colorectal cancer risk perception predict screening behavior? A systematic review and meta-analysis. *Journal of Behavioral Medicine, 38*(6), 837–850. doi: 10.1007/s10865-015-9668-8

Baker, C. W., Little, T. D., & Brownell, K. D. (2003). Predicting adolescent eating and activity behaviors: the role of social norms and personal agency. *Health Psychology* , *22*(2), 189–198. doi: 10.1037/0278-6133.22.2.189

Bonetti, D., & Johnston, M. (2008). Perceived control predicting the recovery of individual-specific walking behaviours following stroke: testing psychological models and

constructs. *British Journal of Health Psychology, 13*, 463–478. doi: 10.1348/135910707X216648
Booth, A. R., Norman, P., Goyder, E., Harris, P. R., & Campbell, M. J. (2014). Pilot study of a brief intervention based on the theory of planned behaviour and self-identity to increase chlamydia testing among young people living in deprived areas. *British Journal of Health Psychology*, *19*(3), 636–651. doi: 10.1111/bjhp.12065
Boucher, D., Gagné, C., & Côté, F. (2015). Effect of an intervention mapping approach to promote the consumption of fruits and vegetables among young adults in junior college: A quasi-experimental study. *Psychology & Health*, *30*(11), 1306–25. doi: 10.1080/08870446.2015.1050393
Brewer, N. T., Chapman, G. B., Gibbons, F. X., Gerrard, M., McCaul, K. D., & Weinstein, N. D. (2007). Meta-analysis of the relationship between risk perception and health behavior: The example of vaccination. *Health Psychology*, *26*(2), 136–145. doi: 10.1037/0278-6133.26.2.136
Broaddus, M. R., Schmiege, S. J., & Bryan, A. D. (2011). An expanded model of the temporal stability of condom use intentions: Gender-specific predictors among high-risk adolescents. *Annals of Behavioral Medicine*, *42*(1), 99–110. doi: 10.1007/s12160-011-9266-0
Brown, S. L., Gibney, T. M., & Tarling, R. (2013). Busy lifestyles and mammography screening: time pressure and women's reattendance likelihood. *Psychology & Health, 28*(8), 928–938. doi: 10.1080/08870446.2013.766734
Cafri, G., Thompson, J. K., Jacobsen, P. B., & Hillhouse, J. (2009). Investigating the role of appearance-based factors in predicting sunbathing and tanning salon use. *Journal of Behavioral Medicine*, *32*(6), 532–544. doi: 10.1007/s10865-009-9224-5
Cavallo, D. N., Brown, J. D., Tate, D. F., DeVellis, R. F., Zimmer, C., & Ammerman, A. S. (2013). The role of companionship, esteem, and informational support in explaining physical activity among young women in an online social network intervention. *Journal of Behavioral Medicine*, *37*(5), 955–966. doi: 10.1007/s10865-013-9534-5
Conner, M., Rhodes, R. E., Morris, B., McEachan, R., & Lawton, R. (2011). Changing exercise through targeting affective or cognitive attitudes. *Psychology & Health*, *26*(2), 133–149. doi: 10.1080/08870446.2011.531570
Conroy, D., Sparks, P., & De Visser, R. (2015). Efficacy of a non-drinking mental simulation intervention for reducing student alcohol consumption. *British Journal of Health Psychology*, *20*(4), 688–707. doi: 10.1111/bjhp.12133
Cooke, R., & French, D. P. (2008). How well do the theory of reasoned action and theory of planned behaviour predict intentions and attendance at screening programmes? A meta-analysis. *Psychology & Health*, *23*(7), 745–765. doi: 10.1080/08870440701544437
Cooper, D. P., Goldenberg, J. L., & Arndt, J. (2014). Perceived efficacy, conscious fear of death and intentions to tan: Not all fear appeals are created equal. *British Journal of Health Psychology, 19*(1), 1–15. doi: 10.1111/bjhp.12019
Craciun, C., Schüz, N., Lippke, S., & Schwarzer, R. (2010). Risk perception moderates how intentions are translated into sunscreen use. *Journal of Behavioral Medicine*, *33*(5), 392–398. doi: 10.1007/s10865-010-9269-5
Darker, C. D., French, D. P., Eves, F. F., & Sniehotta, F. F. (2010). An intervention to promote walking amongst the general population based on an "extended" theory of planned behaviour: a waiting list randomised controlled trial. *Psychology & Health*, *25*(1), 71–88. doi: 10.1080/08870440902893716
Darker, C. D., French, D. P., Longdon, S., Morris, K., & Eves, F. F. (2007). Are beliefs elicited biased by question order? A theory of planned behaviour belief elicitation study

about walking in the UK general population. *Br J Health Psychol*, *12*(1), 93–110. doi: 10.1348/135910706X100458

De Bruijn, G. J., Wiedemann, A., & Rhodes, R. E. (2014). An investigation into the relevance of action planning, theory of planned behaviour concepts, and automaticity for fruit intake action control. *British Journal of Health Psychology*, *19*(3), 652–669. doi: 10.1111/bjhp.12067

De Ven, M. O. M., Van den Eijnden, R., & Engels, R. (2006). Smoking-specific cognitions and smoking behaviour among adolescents with asthma. *Psychology & Health*, *21*(6), 699–716. doi: 10.1080/14768320600603307

de Vries, H., van Osch, L., Eijmael, K., Smerecnik, C., & Candel, M. (2012). The role of risk perception in explaining parental sunscreen use. *Psychology & Health*, *27*(11), 1342–1358. doi: 10.1080/08870446.2012.684059

de Wit, J. B. F., Das, E., & Vet, R. (2008). What works best: Objective statistics or a personal testimonial? An assessment of the persuasive effects of different types of message evidence on risk perception. *Health Psychology*, *27*(1), 110–115. doi: 10.1037/0278-6133.27.1.110

Doshi, A., Patrick, K., Sallis, J. F., & Calfas, K. (2003). Evaluation of physical activity web sites for use of behavior change theories. *Annals of Behavioral Medicine*, *25*(2), 105–111. doi: 10.1207/S15324796ABM2502_06

Drossaert, C. H. C., Boer, H., & Seydel, E. R. (2005). Women's opinions about attending for breast cancer screening: Stability of cognitive determinants during three rounds of screening. *British Journal of Health Psychology*, *10*(1), 133–149. doi: 10.1348/135910704x14645

Epton, T., & Harris, P. R. (2008). Self-affirmation promotes health behavior change. *Health Psychology*, *27*, 746-752. doi:10.1037/0278-6133.27.6.746

Everett, B. G., Rosario, M., McLaughlin, K. A., & Austin, S. B. (2014). Sexual orientation and gender differences in markers of inflammation and immune functioning. *Annals of Behavioral Medicine*, *47*(1), 57–70. doi: 10.1007/s12160-013-9567-6

Farquharson, L., Noble, L. M., Barker, C., & Behrens, R. H. (2004). Health beliefs and communication in the travel clinic consultation as predictors of adherence to malaria chemoprophylaxis. *British Journal of Health Psychology*, *9*(2), 201–217. doi: 10.1348/135910704773891050

Fife-Schaw, C., & Abraham, C. (2009). How much behaviour change should we expect from health promotion campaigns targeting cognitions? An approach to pre-intervention assessment. *Psychology & Health*, *24*(7), 763–76. doi: 10.1080/08870440801956184

Francis, J. J., Johnston, M., Robertson, C., Glidewell, L., Entwistle, V., Eccles, M. P., & Grimshaw, J. M. (2010). What is an adequate sample size? Operationalising data saturation for theory-based interview studies. *Psychology & Health*, *25*(10), 1229–45. doi: 10.1080/08870440903194015

Francis, J. J., Stockton, C., Eccles, M. P., Johnston, M., Cuthbertson, B. H., Grimshaw, J. M., … Stanworth, S. J. (2009). Evidence-based selection of theories for designing behaviour change interventions: Using methods based on theoretical construct domains to understand clinicians' blood transfusion behaviour. *British Journal of Health Psychology*, *14*(4), 625–646. doi: 10.1348/135910708X397025

French, D. P., & Cooke, R. (2012). Using the theory of planned behaviour to understand binge drinking: The importance of beliefs for developing interventions. *British Journal of Health Psychology*, *17*(1), 1–17. doi: 10.1111/j.2044-8287.2010.02010.x

French, D. P., & Hankins, M. (2003). The expectancy-value muddle in the theory of planned behaviour - and some proposed solutions. *British Journal of Health Psychology*, *8*, 37–55. doi: 10.1348/135910703762879192

Gagné, C., & Godin, G. (2007). Does the easy-difficult item measure attitude or perceived behavioural control? *British Journal of Health Psychology*, *12*(4), 543–57. doi: 10.1348/135910706X147781
Gagné, C., & Harnois, I. (2013). Health Psychology The Contribution of Psychosocial Variables in Explaining Preschoolers ' Physical Activity The Contribution of Psychosocial Variables in Explaining Preschoolers ' Physical Activity, *32*(6), 657–665. doi: 10.1037/a0031638
Gaston, A., & Prapavessis, H. (2009). Maternal-fetal disease information as a source of exercise motivation during pregnancy. *Health Psychology* , *28*(6), 726–733. doi: 10.1037/a0016702
Gaston, A., & Prapavessis, H. (2014). Using a combined protection motivation theory and health action process approach intervention to promote exercise during pregnancy. *Journal of Behavioral Medicine*, *37*(2), 173–184. doi: 10.1007/s10865-012-9477-2
Gerend, M. A., Shepherd, J. E., & Monday, K. A. (2008). Behavioral frequency moderates the effects of message framing on HPV vaccine acceptability. *Annals of Behavioral Medicine*, *35*(2), 221–229. doi: 10.1007/s12160-008-9024-0
Gerend, M. A., Shepherd, M. A., & Shepherd, J. E. (2013). The multidimensional nature of perceived barriers: Global versus practical barriers to HPV vaccination. *Health Psychology, 32*(4), 361–369. doi: 10.1037/a0026248
Gibbons, F. X., Houlihan, A. E., & Gerrard, M. (2009). Reason and reaction: The utility of a dual-focus, dual-processing perspective on promotion and prevention of adolescent health risk behaviour. *British Journal of Health Psychology, 14,* 231–248. doi: 10.1348/135910708X376640
Giles, M., McClenahan, C., Armour, C., Millar, S., Rae, G., Mallett, J., & Stewart-Knox, B. (2014). Evaluation of a theory of planned behaviour-based breastfeeding intervention in Northern Irish Schools using a randomized cluster design. *British Journal of Health Psychology*, *19*(1), 16–35. doi: 10.1111/bjhp.12024
Gillibrand, R., & Stevenson, J. (2006). The extended health belief model applied to the experience of diabetes in young people. *British Journal of Health Psychology*, *11*(1), 155–169. doi: 10.1348/135910705X39485
Gratton, L., Povey, R., & Clark-Carter, D. (2007). Promoting children's fruit and vegetable consumption: interventions using the Theory of Planned Behaviour as a framework. *British Journal of Health Psychology*, *12*(4), 639–650. doi: 10.1348/135910706X171504
Guilamo-Ramos, V., Jaccard, J., Dittus, P., & Collins, S. (2008). Parent-adolescent communication about sexual intercourse: an analysis of maternal reluctance to communicate. *Health Psychology, 27*(6), 760–769. doi: 10.1037/a0013833
Hagger, M. S., & Chatzisarantis, N. L. D. (2009). Integrating the theory of planned behaviour and self-determination theory in health behaviour: A meta-analysis. *British Journal of Health Psychology, 14*(2), 275–302. doi: 10.1348/135910708X373959
Hagger, M. S. (2009). Theoretical integration in health psychology: Unifying ideas and complementary explanations. *British Journal of Health Psychology, 14*(2), 189–94. doi: 10.1348/135910708X397034
Hall, S., French, D. P., & Marteau, T. M. (2009). Do perceptions of vulnerability and worry mediate the effects of a smoking cessation intervention for women attending for a routine cervical smear test? An experimental study. *Health Psychology, 28*(2), 258–263. doi: 10.1037/a0013425
Hardcastle, S., Blake, N., & Hagger, M. S. (2012). The effectiveness of a motivational interviewing primary-care based intervention on physical activity and predictors of

change in a disadvantaged community. *Journal of Behavioral Medicine, 35*(3), 318–333. doi: 10.1007/s10865-012-9417-1

Hardeman, W., Johnston, M., Johnston, D. W., Bonetti, D., Wareham, N. J., & Kinmonth, A. L. (2002). Application of the theory of planned behaviour change interventions: A systematic review. *Psychology and Health, 17,* 123-158. doi:10.1080/08870440290013644a

Hardeman, W., Michie, S., Fanshawe, T., Prevost, A. T., Mcloughlin, K., & Kinmonth, A. L. (2008). Fidelity of delivery of a physical activity intervention: Predictors and consequences. *Psychology & Health*, *23*(1), 11–24. doi: 10.1080/08870440701615948

Hardeman, W., Michie, S., Kinmonth, A. L., Sutton, S., on Behalf of the Proactive Project Team. (2011). Do increases in physical activity encourage positive beliefs about further change in the ProActive cohort? *Psychology & Health*, *26*(7), 899–914. doi: 10.1080/08870446.2010.512662

Hardeman, W., Prevost, A. T., Parker, R. A., & Sutton, S. (2013). Constructing multiplicative measures of beliefs in the theory of planned behaviour. *British Journal of Health Psychology*, *18*(1), 122–138. doi: 10.1111/j.2044-8287.2012.02095.x

Hatchell, A. C., Bassett-Gunter, R. L., Clarke, M., Kimura, S., & Latimer-Cheung, A. E. (2013). Messages for men: the efficacy of EPPM-based messages targeting men's physical activity. *Health Psychology* , *32*(2), 24–32. doi: 10.1037/a0030108

Hersey, J. C., Niederdeppe, J., Evans, W., Nonnemaker, J., Blahut, S., Holden, D., … Haviland, M. (2005). The theory of "truth": How counterindustry campaigns affect smoking behavior among teens. *Health Psychology*, *24*(1), 22–31. doi: 10.1037/0278-6133.24.1.22

Hiemstra, M., Otten, R., van Schayck, O. C. P., & Engels, R. C. M. E. (2012). Smoking-specific communication and children's smoking onset: an extension of the theory of planned behaviour. *Psychology & Health*, *27*(9), 1100–17. doi: 10.1080/08870446.2012.677846

Hill, C. A., & Abraham, C. (2007). School-based, randomised controlled trial of an evidence-based condom promotion leaflet. *Psychology & Health*, *23*(1), 41–56. doi: 10.1080/08870440701619726

Hobbs, N., Dixon, D., Johnston, M., & Howie, K. (2012). Can the theory of planned behaviour predict the physical activity behaviour of individuals? *Psychology & Health*, *28*(3), 1–16. doi: 10.1080/08870446.2012.716838

Honda, K., & Gorin, S. S. (2006). A model of stage of change to recommend colonoscopy among urban primary care physicians. *Health Psychology* , *25*(1), 65–73. doi: 10.1037/0278-6133.25.1.65

Huebner, D. M., Neilands, T. B., Rebchook, G. M., & Kegeles, S. M. (2011). Sorting through chickens and eggs: a longitudinal examination of the associations between attitudes, norms, and sexual risk behavior. *Health Psychology* , *30*(1), 110–8. doi: 10.1037/a0021973

Hyde, M. K., & White, K. M. (2013). A test of three interventions to promote people's communication of their consent for organ donation. *Psychology & Health*, *28*(4), 399–417. doi: 10.1080/08870446.2012.731060

Inauen, J., & Mosler, H.-J. (2016). Mechanisms of behavioural maintenance: Long-term effects of theory-based interventions to promote safe water consumption. *Psychology & Health*, *31*(2), 166-183. doi: 10.1080/08870446.2015.1085985

Jacobs, N., Hagger, M. S., Streukens, S., De Bourdeaudhuij, I., & Claes, N. (2011). Testing an integrated model of the theory of planned behaviour and self-determination theory for different energy balance-related behaviours and intervention intensities. *British Journal of Health Psychology*, *16*(1), 113–134. doi: 10.1348/135910710X519305

Janssen, E., van Osch, L., de Vries, H., & Lechner, L. (2011). Measuring risk perceptions of skin cancer: reliability and validity of different operationalizations. *British Journal of Health Psychology*, *16*, 92–112. doi: 10.1348/135910710X514120

Janssen, E., van Osch, L., de Vries, H., & Lechner, L. (2013). Examining direct and indirect pathways to health behaviour: The influence of cognitive and affective probability beliefs. *Psychology & Health*, *28*(5), 546–560. doi: 10.1080/08870446.2012.751108

Janssen, E., van Osch, L., Lechner, L., Candel, M., & de Vries, H. (2012). Thinking versus feeling: Differentiating between cognitive and affective components of perceived cancer risk. *Psychology & Health*, *27*(7), 767–783. doi: 10.1080/08870446.2011.580846

Jemmott, J. B., Jemmott, L. S., O'Leary, A., Ngwane, Z., Icard, L., Bellamy, S., … Makiwane, M. B. (2011). Cognitive-behavioural health-promotion intervention increases fruit and vegetable consumption and physical activity among South African adolescents: a cluster-randomised controlled trial. *Psychology & Health*, *26*(2), 167–85. doi: 10.1080/08870446.2011.531573

Johnston, D. W., Johnston, M., Pollard, B., Kinmonth, A. L., & Mant, D. (2004). Motivation is not enough: prediction of risk behavior following diagnosis of coronary heart disease from the theory of planned behavior. *Health Psychol*, *23*(5), 533–538. doi: 10.1037/0278-6133.23.5.533

Johnston, M., Dixon, D., Hart, J., Glidewell, L., Schröder, C., & Pollard, B. (2014). Discriminant content validity: A quantitative methodology for assessing content of theory-based measures, with illustrative applications. *British Journal of Health Psychology*, *19*(2), 240–257. doi: 10.1111/bjhp.12095

Jones, L. W., Sinclair, R. C., Rhodes, R. E., & Courneya, K. S. (2004). Promoting exercise behaviour: an integration of persuasion theories and the theory of planned behaviour. *British Journal of Health Psychology*, *9*, 505–521. doi: 10.1348/1359107042304605

Jung, M. E., Martin Ginis, K. A., Phillips, S. M., & Lordon, C. D. (2011). Increasing calcium intake in young women through gain-framed, targeted messages: A randomised controlled trial. *Psychology & Health*, *26*(5), 531–47. doi: 10.1080/08870441003611544

Kasparian, N. A., McLoone, J. K., & Meiser, B. (2009). Skin cancer-related prevention and screening behaviors: A review of the literature. *Journal of Behavioral Medicine*, *32*(5), 406–428. doi: 10.1007/s10865-009-9219-2

Lawson, V. L., Bundy, C., Lyne, P. A., & Harvey, J. N. (2004). Using the IPQ and PMDI to predict regular diabetes care-seeking among patients with Type 1 diabetes. *British Journal of Health Psychology*, *9*(2), 241–52. doi: 10.1348/135910704773891078

Lippke, S., & Plotnikoff, R. C. (2009). The protection motivation theory within the stages of the transtheoretical model - stage-specific interplay of variables and prediction of exercise stage transitions. *British Journal of Health Psychology*, *14*(2), 211–229. doi: 10.1348/135910708X399906

Lippke, S., & Plotnikoff, R. C. (2014). Testing two principles of the Health Action Process Approach in individuals with type 2 diabetes. *Health Psychology*, *33*(1), 77–84. doi: 10.1037/a0030182

Lowe, R., Bennett, P., Walker, I., Milne, S., & Bozionelos, G. (2003). A connectionist implementation of the theory of planned behavior: Association of beliefs with exercise intention. *Health Psychology* , *22*(5), 464–70. doi: 10.1037/0278-6133.22.5.464

Mâsse, L. C., Watts, A. W., Barr, S. I., Tu, A. W., Panagiotopoulos, C., Geller, J., & Chanoine, J. P. (2015). Individual and household predictors of adolescents' adherence to a web-based intervention. *Annals of Behavioral Medicine*, *49*(3), 371–383. doi: 10.1007/s12160-014-9658-z

Manning, M., & Bettencourt, B. A. (2011). Depression and medication adherence among breast cancer survivors: Bridging the gap with the theory of planned behaviour. *Psychology & Health*, *26*(9), 1173–1187. doi: 10.1080/08870446.2010.542815

Mark, A. E., Riley, D. L., Mcdonnell, L. A., Pipe, A. L., & Reid, R. D. (2014). Healthy eating opinion survey for individuals at risk for cardiovascular disease, *3Health Psychology, 33*(8), 904–911. doi: 10.1037/a0033525

Marsh, H. W., Papaioannou, A., & Theodorakis, Y. (2006). Causal Ordering of Physical Self-Conceand Exercise Behavior: Reciprocal Effects Model and the Influence of Physical Education Teachers. *Health Psychology*, *25*(3), 316–328. doi: 10.1037/0278-6133.25.3.316

McKenna, F. P., & Horswill, M. S. (2006). Risk taking from the participant's perspective: The case of driving and accident risk. *Health Psychology*, *25*(2), 163–170. doi: 10.1037/0278-6133.25.2.163

Mevissen, F. E. F., Ruiter, R. A. C., Meertens, R. M., & Schaalma, H. P. (2010). The effects of scenario-based risk information on perceptions of susceptibility to Chlamydia and HIV. *Psychology & Health*, *25*(10), 1161–1174. doi: 10.1080/08870440903055901

Mevissen, F. E. F., Meertens, R. M., Ruiter, R. A. C., & Schaalma, H. P. (2012). Bedtime stories: The effects of self-constructed risk scenarios on imaginability and perceived susceptibility to sexually transmitted infections. *Psychology & Health, 27*(9), 1036–1047. doi: 10.1080/08870446.2011.648935

Michie, S., Hardeman, W., Fanshawe, T., Prevost, A. T., Taylor, L., & Kinmonth, L. (2008). Investigating theoretical explanations for behaviour change: The case study of ProActive. *Psychology & Health, 23*(1), 25–39. doi: 10.1080/08870440701670588

Milton, A. C., & Mullan, B. a. (2012). An application of the theory of planned behavior—a randomized controlled food safety pilot intervention for young adults. *Health Psychology, 31*(2), 250–259. doi: 10.1037/a0025852

Molloy, G. J., Dixon, D., Hamer, M., & Sniehotta, F. F. (2010). Social support and regular physical activity: does planning mediate this link? *British Journal of Health Psychology, 15*(4), 859–870. doi: 10.1348/135910710X490406

Montoya, I. D., & Bell, D. C. (2006). Perceiver and relationship effects on perceptions of HIV status: A naturalistic study in a high drug use sample. *Health Psychology*, *25*(6), 771–777. doi: 10.1037/0278-6133.25.6.771

Mullen, P. D., Allen, J. D., Glanz, K., Fernandez, M. E., Bowen, D. J., Pruitt, S. L., … Pignone, M. (2006). Measures used in studies of informed decision making about cancer screening: a systematic review. *Annals of Behavioral Medicine, 32*(3), 188–201. doi: 10.1207/s15324796abm3203_4

Newby, K. V., Wallace, L. M., & French, D. P. (2012). How do young adults perceive the risk of chlamydia infection? A qualitative study. *British Journal of Health Psychology, 17*(1), 144–154. doi: 10.1111/j.2044-8287.2011.02027.x

Newton, J. D., Newton, F. J., & Ewing, M. T. (2014). The dimensional salience solution to the expectancy- value muddle: an extension. *Psychology & Health*, *29*(March), 1458–1475. doi: 10.1080/08870446.2014.950657

Newton, J. D., Ewing, M. T., Burney, S., & Hay, M. (2012). Resolving the theory of planned behaviour's " expectancy-value muddle " using dimensional salience. *Psychology & Health*, *27*(5), 588–602. doi: 10.1080/08870446.2011.611244

Nyembezi, A., Ruiter, R. A. C., van den Borne, B., Sifunda, S., Funani, I., & Reddy, P. (2013). Correlates of consistent condom use among recently initiated and traditionally circumcised men in the rural aread of the Eastern Cape Province of South Africa. *Psychology & Health*, *28*(6), 620-636. doi: 10.1080/08870446.2012.738818

O'Brien, N., Philpott-Morgan, S., & Dixon, D. (2015). Using impairment and cognitions to predict walking in osteoarthritis: A series of n-of-1 studies with an individually tailored, data-driven intervention. *British Journal of Health Psychology*, 52–70. doi: 10.1111/bjhp.12153

O'Carroll, R. E., Dryden, J., Hamilton-Barclay, T., & Ferguson, E. (2011). Anticipated regret and organ donor registration-A pilot study. *Health Psychology , 30*(5), 661–664. doi: 10.1037/a0024182

Ogden, J. (2003). Some problems with social cognition models: a pragmatic and conceptual analysis. *Health Psychology , 22*(4), 424–428. doi: 10.1037/0278-6133.22.4.424

Orbell, S., Perugini, M., & Rakow, T. (2004). Individual differences in sensitivity to health communications: consideration of future consequences. *Health Psychology, 23*(4), 388–396. doi: 10.1037/0278-6133.23.4.388

O'Sullivan, B., McGee, H., & Keegan, O. (2008). Comparing solutions to the "expectancy-value muddle" in the theory of planned behaviour. *British Journal of Health Psychology, 13*(4), 789–802. doi: 10.1348/135910708X278306

Orom, H., Kiviniemi, M. T., Shavers, V. L., Ross, L., & Underwood, W. (2013). Perceived risk for breast cancer and its relationship to mammography in Blacks, Hispanics, and Whites. *Journal of Behavioral Medicine, 36*(5), 466–476. doi: 10.1007/s10865-012-9443-z

Painter, J. E., Borba, C. P. C., Hynes, M., Mays, D., & Glanz, K. (2008). The use of theory in health behavior research from 2000 to 2005: A systematic review. *Annals of Behavioral Medicine*, *35*(3), 358–362. doi: 10.1007/s12160-008-9042-y

Pavey, L. J., & Sparks, P. (2010). Autonomy and reactions to health-risk information. *Psychology & Health*, *25*(7), 885–872. doi: 10.1080/08870440902929528

Payne, N., Jones, F., & Harris, P. R. (2010). A daily diary investigation of the impact of work stress on exercise intention realisation: can planning overcome the disruptive influence of work? *Psychology & Health*, *25*(1), 111–29. doi: 10.1080/08870440903337622

Presseau, J., Tait, R. I., Johnston, D. W., Francis, J. J., & Sniehotta, F. F. (2013). Goal conflict and goal facilitation as predictors of daily accelerometer-assessed physical activity. *Health Psychology*, *32*(12), 1179–87. doi: 10.1037/a0029430

Prestwich, A., Conner, M., Lawton, R., Bailey, W., Litman, J., & Molyneaux, V. (2005). Individual and collaborative implementation intentions and the promotion of breast self-examination. *Psychology & Health, 20,* 743-760. doi:10.1080/14768320500183335

Prestwich, A., Lawton, R., & Conner, M. (2003). The use of implementation intentions and the decision balance sheet in promoting exercise behaviour. *Psychology & Health*, *18*(6), 707–721. doi: 10.1080/08870440310001594493

Quinn, F., Johnston, M., & Johnston, D. W. (2013). Testing an integrated behavioural and biomedical model of disability in N-of-1 studies with chronic pain. *Psychology & Health*, *28*(12), 1391–1406. doi: 10.1080/08870446.2013.814773

Rashidian, A., Miles, J., Russell, D., & Russell, I. (2010). Sample size for regression analyses of theory of planned behaviour studies: Case of prescribing in general practice. *British Journal of Health Psychology*, *11*(4), 581–593. doi: 10.1348/135910705X66043

Reid, A. E., & Aiken, L. S. (2011). Integration of five health behaviour models: common strengths and unique contributions to understanding condom use. *Psychology & Health*, *26*(11), 1499–520. doi: 10.1080/08870446.2011.572259

Rhodes, R. E., & Courneya, K. S. (2003a). Relationships between personality, an extended theory of planned behaviour model and exercise behaviour. *British Journal of Health Psychology, 8,* 19-36. doi: 10.1348/135910703762879183

Rhodes, R. E., & Courneya, K. S. (2003b). Self-efficacy, controllability and intention in the theory of planned behavior: Measurement redundancy or causal independence? *Psychology & Health*, *18*(1), 79–91. doi: 10.1080/0887044031000080665

Rhodes, R. E., & Dickau, L. (2012). Experimental evidence for the intention–behavior relationship in the physical activity domain: A meta-analysis. *Health Psychology, 31*(6), 724–727. doi: 10.1037/a0027290

Rhodes, R. E., & Matheson, D. H. (2005). Discrepancies in exercise intention and expectation: theoretical and applied issues. *Psychology & Health*, *20*(1), 63–78. doi: 10.1080/08870440412331296071

Rhodes, R. E., Plotnikoff, R. C., & Courneya, K. S. (2008). Predicting the physical activity intention-behavior profiles of adopters and maintainers using three social cognition models. *Annals of Behavioral Medicine*, *36*(3), 244–252. doi: 10.1007/s12160-008-9071-6

Rhodes, R. E., Spence, J. C., Berry, T., Deshpande, S., Faulkner, G., Latimer-Cheung, A. E., … Tremblay, M. S. (2015). Predicting changes across 12 months in three types of parental support behaviors and mothers' perceptions of child physical activity. *Annals of Behavioral Medicine*, *49*(6), 853–864. doi: 10.1007/s12160-015-9721-4

Rhodes, R. E., Blanchard, C. M., Benoit, C., Levy-Milne, R., Jean Naylor, P., Symons Downs, D., & Warburton, D. E. R. (2014). Belief-level markers of physical activity among young adult couples: Comparisons across couples without children and new parents. *Psychology & Health*, *29*(11), 1320-1340. doi: 10.1080/08870446.2014.929687

Rhodes, R. E., & Courneya, K. S. (2003). Relationships between personality, an extended theory of planned behaviour model and exercise behaviour. *British Journal of Health Psychology*, *8*(1), 19–36. doi: 10.1348/135910703762879183

Rich, A., Brandes, K., Mullan, B., & Hagger, M. S. (2015). Theory of planned behavior and adherence in chronic illness: a meta-analysis. *Journal of Behavioral Medicine*, *38*(4), 673–688. doi: 10.1007/s10865-015-9644-3

Rivis, A., Abraham, C., & Snook, S. (2011). Understanding young and older male drivers' willingness to drive while intoxicated: the predictive utility of constructs specified by the theory of planned behaviour and the prototype willingness model. *British Journal of Health Psychology*, *16*(2), 445–56. doi: 10.1348/135910710X522662

Rivis, A., Sheeran, P., & Armitage, C. J. (2011). Identification As Determinants of Adolescents' Health Behaviours: Evidence and Correlates. *Psychology & Health*, *26*(9), 1128–1142. doi: 10.1080/08870440903427365

Rivis, A., Sheeran, P., & Armitage, C. J. (2006). Augmenting the theory of planned behaviour with the prototype/willingness model: Predictive validity of actor versus abstainer prototypes for adolescents' health-protective and health-risk intentions. *British Journal of Health Psychology, 11*, 483-500. doi:10.1348/135910705X70327

Robinson, D. H. Z., Perryman, J. P., Thompson, N. J., Amaral, S., & Jacob Arriola, K. R. (2012). Testing the utility of a modified organ donation model among African American adults. *Journal of Behavioral Medicine*, *35*(3), 364–374. doi: 10.1007/s10865-011-9363-3

Schmiege, S. J., Aiken, L. S., Sander, J. L., & Gerend, M. a. (2007). Osteoporosis prevention among young women: psychosocial models of calcium consumption and weight-bearing exercise. *Health Psychology*, *26*(5), 577–87. doi: 10.1037/0278-6133.26.5.577

Scott, S. E., Walter, F. M., Webster, A., Sutton, S., & Emery, J. (2013). The model of pathways to treatment: Conceptualization and integration with existing theory. *British Journal of Health Psychology*, *18*(1), 45–64. doi: 10.1111/j.2044-8287.2012.02077.x

Shi, Y., Ehlers, S., Hinds, R., Baumgartner, A., & Warner, D. O. (2012). Monitoring of exhaled carbon monoxide to promote preoperative smoking abstinence. *Health Psychology*, *32*(6), 714–717. doi: 10.1037/a0029504

Shiloh, S., & Ilan, S. (2005). To test or not to test? Moderators of the relationship between risk perceptions and interest in predictive genetic testing. *Journal of Behavioral Medicine*, *28*(5), 467–479. doi: 10.1007/s10865-005-9017-4

Skår, S., Sniehotta, F. F., Molloy, G. J., Prestwich, A., & Araújo-Soares, V. (2011). Do brief online planning interventions increase physical activity amongst university students? A randomised controlled trial. *Psychology & Health*, *26*(4), 399–417. doi: 10.1080/08870440903456877

Sniehotta, F. F., Gorski, C., & Araujo-Soares, V. (2010). Adoption of community-based cardiac rehabilitation programs and physical activity following phase III cardiac rehabilitation in Scotland: a prospective and predictive study. *Psychology & Health*, *25*(7), 839–854. doi: 10.1080/08870440902915915

Southwell, O., & Fox, J. R. E. (2011). Maternal perceptions of overweight and obesity in children: A grounded theory study. *British Journal of Health Psychology*, *16*(3), 626–41. doi: 10.1348/2044-8287.002002

Stein, J. A., & Nyamathi, A. M. (2010). Completion and subject loss within an intensive hepatitis vaccination intervention among homeless adults: The role of risk factors, demographics, and psychosocial variables. *Health Psychology*, *29*(3), 317–323. doi: 10.1037/a0019283

Strating, M. M. H., Van Schuur, W. H., & Suurmeijer, T. P. B. M. (2006). Contribution of partner support in self-management of rheumatoid arthritis patients. An application of the theory of planned behavior. *Journal of Behavioral Medicine*, *29*(1), 51–60. doi: 10.1007/s10865-005-9032-5

Sweeny, K., & Legg, A. M. (2011). Predictors of interest in direct-to-consumer genetic testing. *Psychology & Health*, *26*(10), 1259–1272. doi: 10.1080/08870446.2010.514607

Sweet, S. N., Ginis, K. a. M., & Latimer-Cheung, A. E. (2012). Examining Physical Activity Trajectories for People With Spinal Cord Injury. *Health Psychology*, *31*(6), 728–732. doi: 10.1037/a0027795

Teasdale, E., Yardley, L., Schlotz, W., & Michie, S. (2012). The importance of coping appraisal in behavioural responses to pandemic flu. *British Journal of Health Psychology*, *17*(1), 44–59. doi: 10.1111/j.2044-8287.2011.02017.x

Tiro, J. A., Diamond, P. M., Perz, C. A., Fernandez, M., Rakowski, W., DiClemente, C. C., & Vernon, S. W. (2005). Validation of scales measuring attitudes and norms related to mammography screening in women veterans. *Health Psychology*, *24*(6), 555–566. doi: 10.1037/0278-6133.24.6.555

Tyson, M., Covey, J., & Rosenthal, H. E. (2014). Theory of planned behavior interventions for reducing heterosexual risk behaviors: A meta-analysis. *Health Psychology*, *33*(12), 1454–1467. doi: 10.1037/hea0000047

van Bree, R. J. H., van Stralen, M. M., Bolman, C., Mudde, A. N., de Vries, H., & Lechner, L. (2013). Habit as moderator of the intention–physical activity relationship in older adults: a longitudinal study. *Psychology & Health*, *28*(5), 514–532. doi: 10.1080/08870446.2012.749476

Van De Ven, M. O. M., Van Den Eijnden, R. J. J. M., & Engels, R. C. M. E. (2006). Smoking-specific cognitions and smoking behaviour among adolescents with asthma. *Psychology & Health*, *21*, 699-716. doi:10.1080/14768320600603307

Van Osch, L., Reubsaet, A., Lechner, L., Candel, M., Mercken, L., & De Vries, H. (2008). Predicting parental sunscreen use: Disentangling the role of action planning in the

intention–behavior relationship. *Psychology & Health*, *23*, 829–847. doi: 10.1080/08870440701596577
Van Zundert, R. M. P., Engels, R. C. M. E., & Van Den Eijnden, R. J. J. M. (2006). Adolescent smoking continuation: Reduction and progression in smoking after experimentation and recent onset. *Journal of Behavioral Medicine*, *29*(5), 435–447. doi: 10.1007/s10865-006-9065-4
Wang, S. H. Q., Borland, R., & Whelan, A. (2005). Determinants of intention to quit: Confirmation and extension of western theories in male Chinese smokers. *Psychology & Health*, *20*(1), 35–51. doi: 10.1080/08870440412331296062
Weinstein, N. D. (2007). Misleading tests of health behavior theories. *Annals of Behavioral Medicine*, *33*(1), 1–10. doi: 10.1207/s15324796abm3301_1
Williams, D. M., Anderson, E. S., & Winett, R. a. (2005). A review of the outcome expectancy construct in physical activity research. *Annals of Behavioral Medicine*, *29*(1), 70–79. doi: 10.1207/s15324796abm2901_10
Wright, A. J., French, D. P., Weinman, J., & Marteau, T. M. (2006). Can genetic risk information enhance motivation for smoking cessation? An analogue study. *Health Psychology* , *25*(6), 740–752. doi: 10.1037/0278-6133.25.6.740
Wyszynski, C. M., Bricker, J. B., & Comstock, B. a. (2011). Parental smoking cessation and child daily smoking: A 9-year longitudinal study of mediation by child cognitions about smoking. *Health Psychology* , *30*(2), 171–176. doi: 10.1037/a0022024

Table B1

Reasons for Exclusion

Theory and study	Reason for exclusion
Theory of Reasoned Action	
Cafri et al. (2009)	Did not test TRA
Cooke & French (2008)	Not an empirical study, meta-analysis
Doshi et al. (2003)	Did not test TRA
Fife-Schaw & Abraham (2009)	Did not test TRA in full form
Gibbons et al. (2009)	Not an empirical article, conceptual review
Newton et al. (2014)	Did not test TRA, tested belief salience
Hersey et al. (2005)	Did not test TRA, multi theory intervention, no correlations available
Ogden (2003)	Not empirical, conceptual review
Robinson et al. (2012)	Did not test TRA, tested other model
Tyson et al. (2014)	Not an empirical study, meta-analysis
Health Belief Model	
Cafri et al. (2009)	Did not test HBM, tested an integrated model but not HBM hypotheses independently
de Vries et al. (2012)	Did not test HBM, focused only on risk perceptions
De Wit et al. (2008)	Did not test HBM, experimental study of risk perceptions and severity
Drossaert et al. (2003)	Did not test HBM, test of TPB
Everett et al. (2014)	Did not test HBM, focused on demographic factors
Gerend et al. (2013)	Did not test HBM, focused only on barriers
Gillibrand & Stevenson (2006)	Did not test HBM, tested an extended HBM whichDid not include traditional hypothesis tests from HBM
Guilamo-Ramos et al. (2008)	Did not test HBM only a generalized model of behavior
Huebner et al. (2011)	Did not test HBM, test of social cognitive predictors
Janssen et al. (2011)	Did not test HBM, only tested risk perceptions
Janssen et al. (2012)	Did not test HBM, focused on cognitive and affective likelihood
Jung et al. (2011)	Did not test HBM, tested an intervention correlations not available
Kasparian et al. (2009)	Not empirical, conceptual review
Lawson et al. (2004)	Did not test HBM, focused on illness beliefs
McKenna & Horswill (2006)	Did not test full HBM, just perceptions of vulnerability
Michie et al. (2008)	Did not test HBM, intervention mapping
Montoya & Bell (2006)	Did not test HBM, test of stereotypes
Mullen et al. (2006)	Not an empirical study, systematic review of measures
Ogden (2003)	Not an empirical study, conceptual review
Orom et al. (2013)	Did not test HBM, only tested risk perceptions
Painter et al. (2008)	Not an empirical article, systematic review
Reid & Aiken (2011)	Did not test HBM, tested an integrated model but not HBM predictions independently

Scott et al. (2013)	Not an empirical article, conceptual review
Southwell & Fox (2009)	Did not test HBM, qualitative research
Stein & Nyamathi (2010)	Did not test full HBM, omitted vulnerability, severity
Sweeny & Legg (2011)	Did not test HBM, only benefits and barriers
Tiro et al. (2005)	Did not test HBM, scale development study
Van Osch et al. (2008)	Did not test HBM, test of HAPA
Protection Motivation Theory	
Albarracin et al. (2003)	Not an empirical study, meta-analysis
Alexander et al. (2014)	Did not test PMT, qualitative study
Atkinson et al. (2015)	Not an empirical study, meta-analysis
Brewer et al. (2007)	Not an empirical study, meta-analysis
Brown et al. (2013)	Did not test PMT, focused only on self-efficacy
Cafri et al. (2009)	Did not test PMT, tested an integrated model but not HBM hypotheses independently
Conroy et al. (2015)	Did not test PMT, experimental study using imagery and implementation intentions
Cooper et al. (2014)	Did not test PMT, experimental test of fear appeals
Craciun et al. (2010)	Did not test PMT, focused on moderation of intention-behaviour by risk perception
de Vries et al. (2012)	Did not test PMT, focused only on risk perceptions
de Wit et al. (2008)	Did not test PMT, experimental study of risk perceptions and severity
Epton & Harris (2008)	Did not test PMT, experimental test of effects of self-affirmation
Gaston & Prapavessis (2009)	Did not test PMT, tested effects of leaflet intervention on PMT variables
Gaston & Prapavessis (2014)	Did not test PMT directly, tested effects of PMT intervention on exercise behavior
Hagger (2009)	Not an empirical study, conceptual review
Hagger et al. (2012)	Did not test PMT, test of SDT and TPB
Hall et al. (2009)	Did not test PMT, focus on vulnerability only
Hatchell et al. (2013)	Did not test PMT, extended parallel-processing model
Janssen et al. (2011)	Did not test PMT, only tested risk perceptions
Janssen et al. (2012)	Did not test PMT, focused on cognitive and affective likelihood
Janssen et al. (2013)	Did not test PMT, examined cognitive and affective probability beliefs
Lippke & Plotnikoff (2009)	Tested PMT under moderator conditions only
Lippke & Plotnikoff (2014)	Did not test PMT, test of HAPA
Mevissen et al. (2010)	Did not test PMT, tested risk information manipulation to change susceptibility perceptions
Mevissen et al. (2012)	Did not test PMT, hypothetical scenarios
Newby et al. (2012)	Did not test PMT, qualitative study
Nyembezi et al. (2007)	Did not test PMT, self-efficacy and susceptibility only
Ogden (2003)	Not an empirical study, conceptual review
Orbell & Hagger (2006)	Did not test PMT, experimental test of temporal framing on health behaviour

Orbell et al. (2004)	Did not test PMT, experimental test of consideration of future consequences effects, correlations not available
Prestwich et al. (2003)	Did not test PMT, experimental study of implementation intentions and decisional balance
Prestwich et al. (2005)	Did not test PMT, implementation intention experiment
Rhodes & Dickau (2012)	Not an empirical study, meta-analysis
Rhodes et al. (2008)	Tested multiple social cognitive models but did report effects on behaviour, correlations not available
Shiloh & Ilan (2005)	Did not test PMT, focus on risk perceptions only
Teasdale et al. (2012)	Did not test PMT, examined threat and advice on perceived severity and intentions
Van Osch et al. (2008)	Did not test PMT, test of HAPA
Weinstein (2007)	Not an empirical study, conceptual review
Williams et al. (2005)	Not an empirical study, measurement review
Wright et al. (2006)	Did not test PMT, experimental study of risk information on motivation to quit smoking
Theory of Planned Behavior	
Abraham et al. (2007)	Did not test TPB, coding techniques in a leaflet
Ajzen (2011)	Not an empirical study, conceptual review
Allom & Mullan (2012)	Did not test TPB, tested effects of habit on behaviour alone
Arden & Armitage (2008)	Included TPB measures in context of intervention and stages of change, correlations not available
Armitage (2004)	Did not test TPB, test of implementation intentions
Armitage (2008)	Did not test TPB, volitional help sheet
Armitage & Arden (2002)	Did not test TPB, tested for discontinuity patterns in transtheoretical model
Armitage & Arden (2008)	Tested TPB in context of intervention and stages of change, correlations not available
Armitage & Reidy (2008)	Tested effects of mental simulations on TPB components, Did not test TPB independently, no correlations available
Armitage et al. (2003)	Did not test TPB, tested for discontinuity patterns in transtheoretical model
Arroll & Howard (2013)	Did not test TPB, qualitative study
Baker et al. (2003)	Did not test full TPB, modified TPB using different conceptualisations of control, correlations not available
Bonetti & Johnston (2008)	Did not test TPB, control perceptions only
Booth et al. (2014)	Included TPB measures in intervention but did not test theory
Boucher et al. (2015)	Included TPB measures in intervention but did not test theory
Broaddus et al. (2011)	Included TPB measures but tested temporal stability and no correlations available
Cavallo et al. (2014)	Did not test TPB, only partial model excluded subjective norms
Conner et al. (2011)	Included TPB measures in intervention but did not test theory predictions and no correlations available
Cooke & French (2008)	Not an empirical study, meta-analysis

Courneya et al. (2010)	Included TPB measures in intervention but did not test theory, correlations not available
Darker et al. (2007)	Did not test TPB, belief elicitation study
Darker et al. (2010)	Included TPB measures in intervention but intervention did not target TPB components separately
De Bruijn et al. (2014)	Did not test TPB predictions, tested predictors of action control
Drossaert et al. (2005)	Did not test TPB predictions, tested changes in TPB variables over course of screening programme
Farquharson et al. (2004)	Did not test TPB, health belief model with intentions and behavioural control
Francis et al. (2009)	Did not test TPB, interview study on theoretical domains
Francis et al. (2010)	Did not test TPB, study on samples sizes for saturation in interview studies
French & Cooke (2012)	Did not test TPB predictions, focused on individual beliefs
French & Hankins (2003)	Did not test TPB, expectancy-value 'muddle' research
Gagné & Godin (2007)	Did not test TPB, tested validity of control measures
Gagné and Harnois (2013)	Did not test full TPB, excluded attitudes and injunctive norms
Giles et al. (2014)	Did not test full TPB, tested as part of intervention and no correlations available
Gratton et al. (2007)	Did not test TPB, part of intervention and no correlations available
Hagger & Chatzisarantis (2009)	Not an empirical study, meta-analysis
Hardcastle et al. (2012)	Did not test TPB in full form, subjective norms omitted
Hardeman et al. (2002)	Not an empirical study, systematic review
Hardeman et al. (2008)	Did not test TPB, intervention fidelity study
Hardeman et al. (2011)	Included TPB measures but tested effects of past behaviour change and no correlations available
Hardemen et al. (2013)	Did not test TPB, belief elicitation study
Hiemstra et al. (2012)	Did not test TPB, used adapted version and omitted intentions
Hill & Abraham (2006)	Did not test TPB, part of intervention and no correlations available
Honda & Gorin (2006)	Did not test TPB, tested integrated model using trans-theoretical model, social cognitive model, and TPB
Hobbs et al. (2014)	Included TPB in context of n-of-1 design, not directly comparable to group-level studies
Hyde & White (2013)	Did not test TPB predictions, tested effects of intention intervention focusing on attitudes and norms
Inauen & Mosler (2016)	Did not test TPB in full form
Jacobs et al. (2011)	Did not test TPB in full form, subjective norms omitted
Jemmott et al. (2011)	Included TPB measures in intervention but intervention did not target TPB components separately
Johnston et al. (2004)	Did not test full TPB, omitted attitudes and subjective norms
Johnston et al. (2014)	Did not test TPB, discriminant content validity
Jones et al. (2004)	Included TPB measures in intervention but did not test theory predictions
Kiviniemi et al. (2007)	Did not test full TPB, omitted intention

Lowe et al. (2003)	Did not test TPB directly, connectionist model, correlations not available
Manning & Bettencourt (2011)	Did not test TPB, used adapted version and correlations not available
Marsh et al. (2006)	Did not test full TPB, omitted subjective norms and did not test mediation of intentions, correlations not available
Mark et al. (2014)	Did not test TPB, belief elicitation study
Mâsse et al. (2015)	Did not test TPB independent of SDT
Michie et al. (2008)	Did not test TPB, behaviour change technique identification case study
Molloy et al. (2010)	Did not test TPB, used adapted version and omitted attitudes
Newton et al. (2012)	Did not test TPB, tested belief salience
O'Brien et al. (2016)	Included TPB measures in n-of-1 design, not directly comparable to group-level studies
O'Carroll et al. (2011)	Included TPB measures in intervention but intervention did not target TPB components separately
Orbell et al. (2004)	Did not test TPB, experimental test of consideration of future consequences effects, correlations not available
O'Sullivan et al. (2008)	Did not test TPB, expectancy-value 'muddle' research
Pavey & Sparks (2010)	used TPB but did not predict intentions independent of other variables, no correlations available
Payne et al. (2010)	used TPB with daily diary design, not directly comparable to group-level studies
Presseau et al. (2013)	Did not test TPB, only effects of intention and PBC on behaviour
Quinn et al. (2013)	Did not test full TPB, used only intentions and perceived behavioural control
Rashidian et al. (2006)	Did not test TPB, examined sample size estimates
Rhodes & Courneya (2003a)	Tested TPB with personality, unique effects not tested and no correlations available
Rhodes & Courneya (2003b)	Did not test TPB, tested dimensionality of perceived behavioural control
Rhodes & Dickau (2012)	Not an empirical study, meta-analysis
Rhodes & Matheson (2005)	Not an empirical study, conceptual review
Rhodes et al. (2007)	Did not test TPB, compared beliefs across illness states
Rhodes et al. (2008)	Did not test TPB independently, tested an integrated model, correlations with intention not available
Rhodes et al. (2014)	Did not test TPB predictions, tested effects of beliefs on behaviour
Rhodes et al. (2015)	Did not test full TPB, used adapted version and omitted subjective norms
Rich et al. (2015)	Not an empirical study, meta-analysis
Rivis et al. (2006)	Tested TPB in experimental context, no correlations without manipulations available
Rivis et al. (2011a)	Did not test full TPB, only predicted behaviour from intentions and identification
Rivis et al. (2011b)	Did not test full TPB, omitted intentions and including prototype willingness model
Schmiege et al. (2007)	Did not test TPB, tested health belief model

Shi et al. (2013)	Did not test TPB, effect of brief advice on smoking abstinence
Skår et al. (2011)	Included TPB measures in intervention but did not test theory
Sniehotta et al. (2010)	Did not test TPB, attitude omitted
Strating et al. (2006)	Did not test full TPB, omitted subjective norms
Sweet et al. (2012)	Included measures of TPB, but tested growth trajectories and no correlations available
Tyson et al. (2015)	Not an empirical study, meta-analysis
van Bree et al. (2013)	Did not test TPB, focused on attitudes-social influences-efficacy (ASE) variables with some TPB variables
Van de Ven et al. (2006)	Did not test full TPB, omitted intention
Van Zundert et al. (2006)	Did not test TPB, omitted intentions
Williams et al. (2005)	Not an empirical study, conceptual review
Wyszynski et al. (2011)	Did not test TPB, focused on parent smoking cessation on child social cognitions
