# Supplementary material for: On Nomological Validity and Auxiliary Assumptions: The Importance of Simultaneously Testing Effects in Social Cognitive Theories Applied to Health Behavior and Some Guidelines
Source: Front Psychol. 2017 Nov 3;8:1933. doi: 10.3389/fpsyg.2017.01933 (PMC5675876; doi:10.3389/fpsyg.2017.01933)
Supplement: Supplementary file 5 [file Appendix_E_Study_Characteristics.pdf]

Appendix E: Characteristics of Studies Included in Illustrative Analysis

Characteristics of Studies Testing Four Social Cognitive Models (Theory of Reasoned Action, Health Belief Model, Protection Motivation Theory, Theory of Planned Behavior)

Theory and study	Claim to test theory (Yes/No)[a]	Claim for support or rejected of the model (Yes/No)[b]	Hypothesized pathway(s) found to be contrary to hypotheses	Falsification hypothesis (Yes/No)	Tested indirect effects	Behavior(s)	Dependent variable	Additional variable(s)/ augmented version of theory[c]
Theory of reasoned action								
Braithwaite et al. (2002)	Yes	No	None	No	No	Genetic testing	Intention	No
Murphy et al. (2014)	Yes	No	Direct effect of subjective norms on behavior	No	Yes	Breast screening	Intention, behavior	Health belief model variables
Zhang et al. (2015)	Yes	Yes, rejected	Direct effect of behavioural beliefs on behavior	No	No	Sexual initiation	Intention, behavior	None
Health belief model								
Byrne et al. (2012)	Yes	No	No effects of perceived benefits, severity and susceptibility on intention	No	No	Influenza vaccination	Intention	Intention, attitude, self-efficacy, personality
Farquharson et al. (2004)	Yes	No	None	No	No	Malaria prophylaxis	Behaviour	Behavioural control, intentions

Gerend & Shepherd (2012)	Yes	No	No effects of perceived severity and benefits on behavior	No	No	HPV vaccination	Behaviour	Theory of planned behaviour variables
Gorin (2005)	Yes	No	No effects of perceived barriers, severity and vulnerability on behavior	No	No	Colorectal cancer screening	Behavior	Intention
Hay et al. (2003)	Yes	No	No effect of perceived severity on behavior	No	No	Colorectal cancer screening	Behavior	Self-efficacy
Jones et al. (2014)	Yes	No	No effect for perceived susceptibility and benefits on behavior	No	No	Food-allergy self-care	Behaviour	Common sense model variables
Kiviniemi et al. (2007)	No	No	No effects of perceived severity and susceptability on behavior	No	No	Physical activity	Behaviour	Theory of planned behaviour variables
Krawczyk et al. (2012)	Yes	Yes, rejected	No effect of perceived severity on behavior	No	No	HPV vaccination	Behavior	Theory of planned behaviour variables
Manne et al. (2002)	No	No	No effect of perceived severity on screening attendance	No	No	Colorectal cancer screening	Behavior	Trans-theoretical model variables
Montanaro & Bryan (2014)	Yes	No	No effects for perceived susceptibility, severity, benefits, and barriers on behavior	No	No	Condom use	Behaviour	Theory of planned behaviour variables
Murphy et al. (2014)	Yes	No	Perceived benefits had no effect on behavior	No	Yes	Breast screening	Behaviour	Theory of planned

								behaviour variables
Norman & Brain (2005)	Yes	No	None	No	No	Breast self-examination	Behavior	Self-efficacy
Schmiege et al. (2007)	Yes	Yes, rejected	No effects for perceived susceptibility, severity and benefits on calcium consumption (sample 1, time 1 and time 2; sample 2, time 2) and exercise behaviour (sample 1, time 1 and time 2; sample 2, time 1), no effects of perceived severity on calcium consumption (sample 2, time 1), and no effect of perceived susceptibility and benefits on exercise behaviour (sample 2, time 2)	No	No	Weight-bearing physical activity and calcium consumption	Behavior	Intention
Wang et al. (2007)	Yes	No	No effects for perceived susceptibility, severity, and barriers on genetic testing decisions	No	No	Genetic testing	Behavior	Worry
Protection motivation theory								
Boer & Mashamba (2007)	Yes	Yes, supported	No effects for perceived susceptibility, vulnerability, and fear on condom use intentions (protection motivation) in male and female samples	No	No	Condom use	Intention, behaviour	Theory of planned behaviour variables
Ch’ng & Glendon (2014)	Yes	Yes, supported	None	No	Yes	Sun protection	Protection motivation	None

						behaviors	, behaviour	
Milne et al. (2002)	Yes	Yes, supported	None	No	No	Physical activity	Intention, behavior	None
Norman et al. (2003)	Yes	Yes, supported	No effect of severity on protection motivation	No	No	Eye patch use in amblyopia	Protection motivation , behavior	None
Orbell et al. (2009)	No	No	No effect of susceptibility, fear, negative outcome expectancy, and self-efficacy on intentions (protection motivation) to not smoke	No	No	Smoking	Intention, behaviour	Theory of planned behaviour, social cognitive theory, and health action process approach variables
Plotnikoff et al. (2010)	Yes	Yes, supported	No effect for perceived vulnerability on physical activity intentions (protection motivation) in both samples and no effect for severity on intentions (protection motivation) in the type II diabetes sample	No	No	Physical activity	Intention, behavior	None
Plotnikoff et al. (2014)	Yes	No	No effects of severity, vulnerability, and self-efficacy on intention (protection motivation)	No	No	Physical activity	Intention, behavior	Theory of planned behaviour and social cognitive theory variables

Smerecnik et al. (2011)	Yes	No	No effect of response costs on intention (protection motivation)	No	No	Genetic testing for smoking addiction	Intention	None
Tulloch et al. (2009)	Yes	Yes, supported	No effect for perceived vulnerability on physical activity intentions (protection motivation) for both short- and long-term behavioral prediction	No	No	Physical activity	Intention, behavior	None
Umeh (2004)	Yes	No	No effect of threat appraisals (severity, vulnerability) and coping appraisals (self-efficacy) on condom use intentions (protection motivation)	No	No	Condom use	Intention	None
Theory of planned behavior								
Abraham & Sheeran (2004)	No	Yes, supported	None	No	No	Physical activity	Intention	Anticipated regret
Abraham et al. (2004)	No	No	No effect of perceived behavioral control on intentions	No	No	Condom use	Intention, behavior	Descriptive norm, partner approval, anticipated regret
Araujo-Soares et al. (2013)	Yes	No	None	No	No	Sunscreen use	Intention, behavior	Prototype similarity, descriptive norms, planning
Arbour-Nicitopoulos et	Yes	Yes,	None	No	No	Physical	Intention,	Neighbourh

al. (2010)		supported				activity	behavior	ood aesthetics, sidewalk presence
Armitage (2005)	Yes	Yes, supported	No effect of attitudes on intentions and no effect of intentions on behavioural maintenance	No	No	Physical activity	Intention, behavior	None
Armitage et al. (2002)	Yes	No	No effect of perceived behavioral control on intention (condom use sample only)	No	No	Condom use, binge drinking, drink driving, and health screening	Intention, behaviour	Multidimen sional health locus of control
Bagot et al. (2015)	Yes	No	No effect of attitudes on blood donation intentions (distant donors sample)	No	No	Changing type of blood donated	Intention	Moral norm, anticipatory regret, donor identity
Boer & Mashamba (2007)	Yes	Yes, supported	No effects of perceived behavioural control on intentions (male sample) and no effect of perceived behavioural control and subjective norms on intentions (female sample)	No	No	Condom use	Intention	Protection motivation theory variables
Bonetti et al. (2009)	Yes	Yes, supported	No effect of subjective norms and perceived behavioural control on intentions	No	No	Dentists use of fissure sealants	Intention, behavior	Social cognitive theory, common sense

								model, operant learning theory, action planning, and precaution adoption process model variables
Booth et al. (2004)	Yes	No	None	No	No	Chlamydia testing	Intention	Descriptive norm
Brickell et al. (2006)	Yes	Yes, supported	No effect of subjective norms on intentions	No	No	Physical activity	Intention, behavior	Implementa tion intentions
Browne & Chan (2012)	Yes	Yes, supported	None	No	No	Communica tion about mammogra phy	Intnetion, behavior	Implementi on intention
Bryan et al. (2002)	Yes	Yes, supported	No effect of perceived behavioural control on intention (Study 1 only)	No	Yes	Condom use	Intention, behavior	None
Chan et al. (2014)	Yes	Yes, supported	None	No	Yes	Myopia preventive behaviour	Intention, behaviour	Self-determinati on theory variables
Chan et al. (2015)	No	No	None	No	Yes	Wearing facemasks to prevent H1N1	Intention, behaviour	Self-determinati on theory

Chatzisarantis & Hagger (2008)	No	No	None	No	No	Physical activity	Intention, behavior	Continuation intentions, personality
Churchill & Jessop (2011)	Yes	No	None	No	No	Dietary behaviour	Intention, behavior	Impulsivity, self-control
Conner & Godin (2007)	No	No	No effect of subjective norm on intention in all samples and no effect of attitude on intentions in sample 2 (quitting smoking) and 3 (exercise)	No	No	Looking after health; physical activity; quitting smoking;	Intention, behavior	Intention stability
Conner et al. (2002)	Yes	Yes, supported	No effect of subjective norms on intentions (healthy eating) and no effect of intention on behaviour (fiber intake, eating fruit and vegetables)	No	No	Healthy eating, fat intake, dietary fiber intake, eating fruit and vegetables	Intention, behavior	Intention stability
Conner et al. (2006)	Yes	No	None	No	No	Smoking	Intention, behavior	Anticipated regret
Conner et al. (2008)	Yes	No	No effect for subjective norms in all samples, no effect for affective attitudes in male samples, no effect for cognitive attitudes in female samples	No	No	Condom use	Intention	Intoxication, arousal
Conner et al. (2010)	Yes	Yes, supported	No effect of subjective norms on intention (sample 2)	No	No	Physical activity	Intention, behavior	Action planning
Conner et al. (2013)	No	No	None	No	No	Blood donation	Intention, behavior	Anticipated affective reactions

Conner et al. (2015)	No	No	None	No	No	Health-related behaviors (N = 20)	Intention, behavior	Anticipated affective reactions
Cooke & French (2011)	No	No	No effect of perceived behavioural control on intentions (bar sample) and no effect of subjective norms on intentions (library sample)	No	No	Alcohol consumptio n	Intention	None
Courneya et al. (2002)	No	No	No effect of attitude and perceived behavioural control on intentions	No	No	Physical activity	Intention, behavior	Personality
Courneya et al. (2006)	Yes	No	No effect of subjective norms (injunctive norms) on intention	No	No	Physical activity	Intention, behavior	None
De Bruijn et al. (2007)	No	No	None	No	No	Eating fruit and vegetables	Intention, behavior	Habit
De Bruijn et al. (2009)	No	No	No effect for subjective norms on fruit consumption intentions	No	No	Eating fruit	Intention, behavior	Personality
Drossaert et al. (2003)	Yes	No	None	No	No	Breast screening	Intention, behavior	None
Gardner et al. (2012)	No	No	No effect of subjective norms on binge drinking intentions	No	No	Binge drinking	Intention, behavior	Habit, self-identity
Gerend & Shepherd (2012)	Yes	Yes, supported	None	No	No	HPV vaccination	Intention, behavior	Health belief model variables
Gredig et al. (2006)	Yes	Yes, supported	No effect of subjective norms on intentions	No	No	Condom use	Intention, behavior	None
Hagger at al. (2002)	No	No	None	No	Yes	Physical activity	Intention	Self-determinati on theory

								variables
Hagger et al. (2009)	Yes	Yes, supported	None	No	Yes	Physical activity	Intention, behavior	Self-determination theory variables
Hagger et al. (2012)	Yes	Yes, supported	None	No	Yes	Alcohol consumption	Intention, behavior	Self-determination theory variables
Hanbury et al. (2010)	No	No	No effect of attitudes and perceived behavioural control on intentions	No	No	Adherence to mental health guidelines	Intention, behavior	None
Hardeman et al. (2011)	Yes	Yes, rejected	No effect of subjective norms on intentions and no effect of intentions on behaviour	No	No	Physical activity	Intention, behavior	None
Hassandra et al. (2012)	Yes	No	No effect of subjective norms on intentions (elementary school sample)	No	No	Smoking	Intention	Self-identity, parental attitudes
Hukkelberg et al. (2014)	No	No	None	No	No	Smoking	Intention	None
Hunter et al. (2003)	No	No	None	No	No	Visit GP for breast symptoms	Intention	Common sense model variables
Huston et al. (2010)	No	No	None	No	No	Hormone therapy use	Intention	None
Hyde & White (2009)	Yes	Yes, supported	No effect of perceived behavioural control (control) beliefs on intention to discuss donation	No	No	Registering and discussing organ	Intention	None

						donation		
Hyde & White (2010)	Yes	No	None	No	No	Registering and discussing organ donation	Intention	Prototype willingness model variables
Inauen et al. (2014)	No	No	No effect of subjective norms and perceived behavioural control on intention	No	No	Switching to arsenic-free wells	Intention, behavior	Descriptive norm, commitment strength
Jellema et al. (2013)	Yes	No	No effect of perceived behavioural control on intentions and intentions on behaviour (recommendation-only sample) and no effect of intention on behaviour (recommendation-plus-referral sample)	No	No	Condom use	Intention, behavior	None
Johnston & White (2003)	Yes	Yes, supported	None	No	No	Binge drinking	Intention, behavior	Group norms
Jones et al. (2005)	No	No	No effect of intention on behaviour for recommendation + referral group	No	No	Physical activity recommendations	Intention, behavior	None
Kassem & Lee (2004)	Yes	Yes, supported	None	No	No	Soft drink consumption	Intention	None
Kor & Mullan (2011)	Yes	No	No effect of attitudes on intentions and no effect of intentions on behaviour	No	No	Sleep hygiene	Intention, behavior	Perceived autonomy support, response inhibition
Kothe & Mullan (2015)	Yes	Yes,	None	No	No	Eating fruit	Intention,	None

		supported				and vegetables	behavior	
Krawczyk et al. (2012)	Yes	No	No effect of perceived behavioural control on intentions	No	No	HPV vaccination	Intention, behavior	Health belief model variables
Latimer & Martin Ginis (2005)	No	No	None	No	No	Physical activity	Intention	Fear of negative evaluation
Lawton et al. (2012)	Yes	No	No effect of perceived behavioural control (perceived behavioural control and self-efficacy) and subjective norms (injunctive norms) on intentions (sample 1 only: White British)	No	No	Breast feeding	Intention, behaviour	Moral norms
Legare et al. (2003)	Yes	No	No effect of perceived behavioral control on intentions (perimenopausal sample only)	No	No	Adherence to hormone replacement therapy	Intention	Moral norm
Matterne et al. (2011)	No	No	No effect of subjective norms on behavior	No	No	Skin protection behaviour	Intention, behavior	Prototype willingness model and health action process approach variables
McMillan et al. (2008)	No	No	None	No	No	Breast feeding	Intention, behaviour	Descriptive norm, moral norm, self-identity

McMillan et al. (2009)	No	No	No effect of subjective norms on intentions (formula feeding only)	No	No	Breast feeding and formula feeding infants	Intention, behaviour	No
Mercken et al. (2011)	Yes	No	None	No	Yes	Smoking	Intention, behavior	Future friends' norms
Michie et al. (2004)	No	No	None	No	No	Genetic screening	Intention, behavior	None
Milton & Mullan (2012)	Yes	No	No effect of attitudes and subjective norms on intentions	No	No	Hygienic food preparation	Intention, behavior	None
Moan & Rise (2006)	Yes	Yes, rejected	No effect of intention on behaviour	No	No	Smoking	Intention, behavior	Action planning, moral norms, self-identity
Moan et al. (2005)	Yes	No	No effect of attitudes on intentions	No	No	Parental smoking in front of children	Intention	Moral norms, smoker identity, parent identity, anticipated affect
Montanaro & Bryan (2014)	Yes	Yes, supported	None	No	No	Condom use	Intention, behaviour	Health belief model variables
Morrison et al. (2010)	Yes	No	No effect of subjective norms on intentions	No	No	Cannabis use	Intention, behavior	Experience, distress,

								adversity, and social network use
Moser & Aiken (2011)	No	No	No effect of perceived behavioural control on intentions (Sample 1 only)	No	Yes	Getting breast implants	Intention	Anticipated regret, descriptive norms, image norms, preparatory steps, perceived susceptability and severity
Murnaghan et al. (2010)	Yes	Yes, supported	None	No	Yes	Physical activity; smoking; eating fruit and vegetables	Intention, behavior	None
Newton et al. (2013)	No	No	None	No	No	Organ donation and condom use	Intention	Moral norms, anticipated regret, personal norm
Norman & Conner (2006)	Yes	No	No effect of subjective norms on intentions	No	No	Binge drinking	Intention, behavior	None
Norman & Cooper (2011)	Yes	No	No effect of attitudes on intentions and no effect of intentions on behaviour	No	No	Breast self-examination	Intention, behavior	Past behaviour, context

								stability, habit strength
Orbell & Hagger (2006a)	No	No	None	No	No	Physical activity	Intention	Consideration of future consequences
Orbell & Hagger (2006b)	Yes	No	None	No	No	Colposcopy clinic attendance	Intention, behaviour	Reactance
Orbell et al. (2006)	Yes	No	None	No	No	Colposcopy clinic attendance	Intention, behavior	Illness perceptions
Orbell et al. (2009)	No	No	No effect of attitudes and perceived behavioural control (general self-efficacy) on intentions	No	No	Smoking	Intention, behavior	Protection motivation theory, social cognitive theory, and health action process approach variables
Payne et al. (2004)	No	No	No effect of cognitive attitudes on intentions (physical activity sample only)	No	No	Physical activity and healthy eating	Intention, behavior	Perceived need
Payne et al. (2005)	Yes	No	No effect of subjective norms on intentions (all behaviours), no effect of affective attitudes on intentions (sweets and snack	No	No	Physical activity, eating sweets and	Intention, behavior	Job strain

			foods) and no effect for cognitive attitudes on intentions (physical activity, eating fruit and vegetables)			snack foods, eating fruit and vegetables		
Plotnikoff et al. (2010)	Yes	Yes, supported	None	No	Yes	Physical activity	Intention, behavior	None
Plotnikoff et al. (2014)	Yes	No	No effects of subjective norms on intentions	No	No	Physical activity	Intention, behavior	Protection motivation theory and social cognitive theory variables
Prati et al. (2014)	Yes	No	No effect of subjective norms and perceived behavioural control on intentions	No	No	Condom use	Intention, behavior	None
Presseau et al. (2011)	No	No	No effect of subjective norms on intention	No	No	Physical activity	Intention, behavior	Goal conflict, goal facilitation
Rhodes & Blanchard (2008)	Yes	No	No effect of subjective norms on intentions (community sample) and no effect of attitudes and subjective norms (student sample)	No	Yes	Physical activity	Intention, behavior	
Rhodes et al. (2006)	Yes	No	No effect of subjective norms (injunctive norms) and perceived behavioral control (skills, opportunity, resources) on intentions; Direct effect of attitudes on behaviour	No	Yes	Physical activity	Intention, behavior	None
Rhodes et al. (2014)	Yes	Yes,	No effect of subjective norms on	No	No	Physical	Intention,	None

		supported	intentions (wives only)			activity	behavior	
Rise et al. (2008)	Yes	Yes, rejected	No effect of attitudes (instrumental) and subjective norms on intentions and no effect of intentions on behaviour	No	No	Smoking	Intention, behavior	Descriptive norms
Rivis & Sheeran (2003)	No	No	None	No	No	Physical activity	Intention, behavior	Descriptive norms, prototype similarity, prototype evaluation
Sainsbury et al. (2015)	Yes	No	No effect of subjective norms and perceived behavioural control on intentions	No	No	Eating gluten-free diet	Intention, behavior	None
Scott et al. (2007)	Yes	No	No effect of attitudes and subjective norms on intentions (Study 1), no effect of attitudes on intentions and no effect of intentions on behaviour (Study 2)	No	No	Physical activity	Intention, behavior	None
Siverding et al. (2009)	No	No	None	No	No	Cancer screening	Intention, behavior	Descriptive norms
Steadman & Rutter (2004)	No	No	Direct effect of subjective norms on intention	No	No	Breast screening	Intention, behavior	Modal and ranked beliefs
Steadman et al. (2002)	No	No	None	No	Yes	Breast screening	Intention, behavior	Individual and model normative beliefs
Symons Down & Hausenblas (2007)	Yes	Yes, supported	No effect of attitudes and perceived behavioural control on intentions	No	No	Physical activity	Intention, behavior	None

Taylor et al. (2004)	Yes	Yes, rejected	No effect of attitudes on intentions (for males) and no effect of attitudes and subjective norms on intentions (for females)	No	Yes	Regulation of blood pressure	Intention	Desire, anticipated emotions, trying
Tessier et al. (2015)	No	No	No effect of attitudes on intentions	No	No	Physical activity	Intention, behavior	None
Umeh & Patel (2004)	No	Yes, supported	No effect of perceived behavioural control on intentions	No	No	Ecstasy use	Intention	None
Vallance et al. (2008)	No	No	None	No	No	Physical activity	Intention, behavior	Planning, descriptive norm
Van den Putte et al. (2009)	No	No	None	No	No	Smoking	Intention, behavior	Smoking-identity, quitting identity
Van de Ven et al. (2007)	No	No	Direct effects of subjective norms and attitudes on intentions	No	No	Smoking (asthmatics and non-asthmatics)	Intention, behavior	None
Van Lettow et al. (2015)	No	No	None	No	No	Alcohol consumptio n	Intention, behavior	Prototype similarity, prototype favorability
Walsh et al. (2005)	Yes	No	No effect of attitudes on intentions	No	No	Participatin g in exercise videos	Intention, behavior	Self-concordanc e, conscientio usness
White et al. (2008)	Yes	Yes, supported	None	No	No	Sun-protective behaviours	Intention, behavior	Group norm, image norm

White et al. (2015)	Yes	No	No effect of perceived behavioural control on intentions	No	No	Sun protective behaviors	Intention, behavior	Group norm, image norm, personal norm
Whitford & Jones (2011)	No	No	No effect of intentions on behaviour	No	No	Pelvic-floor exercise	Intention, behavior	Cues ot action
Wilkinson & Abraham (2004)	No	No	None	No	No	Smoking	Intention, behavior	Personality, self-esteem
Wolff et al. (2010)	No	No	No effect of perceived behavioural control on intentions	No	No	Genetic testing	Intention, behavior	Uncertainty avoidance
Wong & Mullan (2009)	Yes	No	No effect of subjective norms on intentions	No	No	Breakfast consumptio n	Intention, behavior	Executive function
Zimmerman & Sieverling (2010)	Yes	No	No effect of attitudes (both samples) and subjective norms (males only) on intentions	No	No	Alcohol consumptio n (males and females)	Intention, behavior	Prototype willingness model variables

Note. [a]Explicit statement of an aim or hypothesis to test the full theory in its common form; [b]Explicit statement that the data provide support for the model or have led to its rejection/falsification. [c]Past behaviour, self-efficacy, belief measures, and demographic variables are not listed as an additional variables as they are considered variables routinely collected, and controlled for, in research on social cognitive theories or are considered akin to existing constructs.
